# An individualized stemness‐related signature to predict prognosis and immunotherapy responses for gastric cancer using single‐cell and bulk tissue transcriptomes

**DOI:** 10.1002/cam4.6908

**Published:** 2024-01-03

**Authors:** Linyong Zheng, Jingyan Chen, Wenhai Ye, Qi Fan, Haifeng Chen, Haidan Yan

**Affiliations:** ^1^ Fujian Key Laboratory of Medical Bioinformatics, Department of Bioinformatics, School of Medical Technology and Engineering Fujian Medical University Fuzhou China; ^2^ Department of Gastrointestinal Surgery Fuzhou Second Hospital Fuzhou China; ^3^ Key Laboratory of Ministry of Education for Gastrointestinal Cancer, The School of Basic Medical Sciences Fujian Medical University Fuzhou China

**Keywords:** gastric cancer, immunotherapy responses, individualized signature, prognosis, stemness

## Abstract

**Background:**

Currently, many stemness‐related signatures have been developed for gastric cancer (GC) to predict prognosis and immunotherapy outcomes. However, due to batch effects, these signatures cannot accurately analyze patients one by one, rendering them impractical in real clinical scenarios. Therefore, we aimed to develop an individualized and clinically applicable signature based on GC stemness.

**Methods:**

Malignant epithelial cells from single‐cell RNA‐Seq data of GC were used to identify stemness‐related signature genes based on the CytoTRACE score. Using two bulk tissue datasets as training data, the enrichment scores of the signature genes were applied to classify samples into two subtypes. Then, using the identified subtypes as criteria, we developed an individualized stemness‐related signature based on the within‐sample relative expression orderings of genes.

**Results:**

We identified 175 stemness‐related signature genes, which exhibited significantly higher AUCell scores in poorly differentiated GCs compared to differentiated GCs. In training datasets, GC samples were classified into two subtypes with significantly different survival times and genomic characteristics. Utilizing the two subtypes, an individualized signature was constructed containing 47 gene pairs. In four independent testing datasets, GC samples classified as high risk exhibited significantly shorter survival times, higher infiltration of M2 macrophages, and lower immune responses compared to low‐risk samples. Moreover, the potential therapeutic targets and corresponding drugs were identified for the high‐risk group, such as *CD248* targeted by ontuxizumab.

**Conclusions:**

We developed an individualized stemness‐related signature, which can accurately predict the prognosis and efficacy of immunotherapy for each GC sample.

## Graphical abstract

By integrating single‐cell RNA‐Seq data and bulk tissue data, we developed a qualitative stemness‐related signature, 47 gene pairs, which can accurately predict patients' prognosis and immunotherapy responses at an individual level. This makes it applicable to real clinical scenarios and assists doctors in formulating appropriate treatment plans for gastric cancer patients.

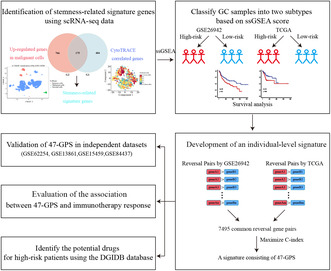

## 1 BACKGROUND

Gastric cancer (GC) remains a prevalent disease globally.^1^ Surgical resection, chemotherapy, and immunotherapy are commonly used treatments for GC. However, nearly 36%–45% of GC patients experience relapse after curative gastrectomy,^2^, ^3^ and about 34% of patients are resistant to chemotherapy.^4^ Only about 26% of patients could benefit from chemotherapy combined with immunotherapy.^5^ Therefore, it is essential to develop a clinically applicable molecular marker to predict prognosis and guide clinical therapy.

Cancer stem cells have attracted a lot of attention for their role in tumorigenesis, metastasis, and drug resistance.^6^, ^7^ In 2018, Malta et al. developed a mRNA expression based‐index (mRNAsi) model using machine learning algorithm to evaluate the stemness of tumors.^8^ Based on mRNAsi, many stemness signatures have been constructed for prognostic analysis,^9^, ^10^, ^11^ including GC.^12^, ^13^, ^14^ However, the absolute stemness index calculated by mRNAsi is easily influenced by sample composition,^15^ and in some cancers, including GC, contrary to previous research, higher stemness corresponds to a better prognosis.^15^, ^16^, ^17^, ^18^ Except for mRNAsi‐based models, other stemness‐related prognostic models were also developed for GC based on known stemness genes. All these models were evaluated using risk scores calculated by the weighted expression values of signature genes, utilizing different thresholds, such as mean, median, and optimal thresholds, to classify patients into different risk groups.^19^, ^20^ Here, we collectively referred to these stemness‐related prognostic models as the threshold‐based scoring models. These models used different thresholds for different datasets, even when the same model is applied. Due to batch effects, we cannot tell which threshold is accurate for a patient who needs to predict prognosis in the clinic. Therefore, these models are not applicable in real clinical scenarios. Previous studies have demonstrated that the within‐sample relative expression orderings (REOs) of genes are robust to batch effects,^21^ and could be used for individualized analysis.^22^, ^23^ Thus, it is worthwhile to try to identify an individualized stemness‐related prognostic signature by the REO‐based method.

In this study, we used malignant epithelial cells from a single‐cell RNA‐Seq (scRNA‐Seq) dataset to identify stemness‐related signature genes based on the CytoTRACE scores, and evaluated them in an external independent scRNA‐Seq dataset. Then, single‐sample gene set enrichment analysis (ssGSEA) was applied to evaluate the enrichment scores of these stemness‐related signature genes for each bulk tissue sample in training data, and the samples were grouped into two subtypes. Using the two subtypes as criteria, we further developed an individualized REO‐based signature, which can predict the prognosis and immunotherapy outcomes of GC samples.

## 2 MATERIALS AND METHODS

### 2.1 Preprocessing of bulk tissue transcriptomes

As shown in Table 1, the bulk tissue transcriptomes of GC with survival information were collected from publicly available sources, including the Gene Expression Omnibus (GEO) and The Cancer Genome Atlas (TCGA) databases. For the microarray data from GEO, the mean expression signals of probes within a gene is its expression value. For the data from TCGA, the Level 3 data were downloaded by R package TCGAbiolinks, and ENSEMBL ID was changed to Entrez gene ID. The clinical information of GC samples in TCGA was also collected using R package RTCGA.clinical. The TCGA and Asian Cancer Research Group (ACRG) classified GC samples into four subtypes, the related subtype information was obtained from a published article by Pan et al.^24^

**TABLE 1:** The training and testing data.

Dataset	Platform	Gastric cancer samples
Training data		
GSE26942^a^	Illumina GPL6947	202
TCGA^a^	RNA‐Seq	375
Testing data		
GSE62254^a^	Affymetrix GPL570	300
GSE15459	Affymetrix GPL570	200
GSE84437	Illumina GPL6947	433
GSE13861^a^	Illumina GPL6884	90

Abbreviations: GSE, gene set enrichment; TCGA, The Cancer Genome Atlas.

^a^ Indicates that the corresponding datasets have not only OS information, but also DFS/RFS information.

### 2.2 Preprocessing of scRNA‐Seq data

The cells of scRNA‐Seq data (GSE134520), from the Tumor Immune Single Cell Hub 2 database (http://tisch.comp‐genomics.org/), were annotated into malignant cells, stromal cells, and immune cells.^25^ For the data of HRA002108, the FASTQ files were collected from the National Genomics Data Center database,^26^ and were processed by Cell Ranger (version 6.0.1) using the GRCh38 genome as reference. We then transformed the result of Cell Ranger into Seurat objects (Seurat v4.3.0).^27^ After cell filtration and normalization, batch effect correction was performed by Harmony (version 0.1.1).^28^ The top 2000 highly variable genes were used for principal component analysis, and 15 principal components were detected. The main cell types were annotated by the expressions of curated known cell markers.^26^ Differentially expressed genes were detected by the “FindMarkers” function (log2 fold change >0.25, and FDR <0.05). The CytoTRACE scores were calculated to estimate the stemness of epithelial cells.^29^

### 2.3 Calculation of enrichment scores

The enrichment score of stemness‐related signature genes for each bulk tissue was evaluated by the “GSVA” package.^30^ The enrichment score for a cell was calculated by the AUCell.^31^

### 2.4 Identification of gene pairs with significantly stable REOs

In a sample, the REOs of a gene pair (*i* and *j*) are either represented as $E_{i}>E_{j}$ or $E_{i}<E_{j}$, where $E_{i}$ and $E_{j}$ are the expressions of gene *i* and *j*, respectively. For GC samples within a subtype (high risk or low risk), we identified gene pairs with stable REOs by the cumulative binomial distribution model:

P=1−∑i=0s−1kiPei1−Pek−i.

Here, *k* is the number of samples within a subtype, *s* is the number of samples with the REO pattern ($E_{i}>E_{j}$ or $E_{i}<E_{j}$), and *P*_*e*_ is 0.5.

### 2.5 Development of an individualized stemness‐related signature

Based on the enrichment scores of stemness‐related signature genes using ssGSEA, the GC subtypes, including high‐risk group and low‐risk group, were identified in TCGA and GSE26942, respectively. Then, the stable gene pairs were separately identified in two subtypes (FDR <5%). The stable gene pairs shared in both two subtypes, but with reversal REO patterns ($E_{i}>E_{j}$→$E_{i}<E_{j}$ or $E_{i}<E_{j}$→$E_{i}>E_{j}$), were selected in TCGA and GSE26942, respectively. And, the common reversal gene pairs of TCGA and GSE26942 were further detected. For a reversal gene pair (*i* and *j*), if its stable REO pattern was $E_{i}>E_{j}$ in high‐risk group and $E_{i}<E_{j}$ in low‐risk group, a GC sample would be classified as high risk if its REO pattern was $E_{i}>E_{j}$; otherwise, low risk. Based on this rule, the common reversal gene pairs significantly correlated with overall survival (OS), were set as candidate prognostic gene pairs by univariate cox regression analysis in pooled data of TCGA and GSE26942 with samples treated surgery alone. We calculated the C‐index value for every candidate gene pair, and all pairs were ranked in descending order according to their C‐index values. Based on the forward search algorithm, we optimized a set of prognostic gene pairs achieving the largest C‐index value as individualized signature using the half voting rule. The half voting rule: a GC would be classified into high‐risk group if at least half of signature gene pairs in this GC supported for high risk; otherwise, low risk.

### 2.6 The TME score and macrophage cell fraction analysis

Using the bulk tissue transcriptomes of GCs, the stromal, immune, and ESTIMATE scores, and tumor purity were evaluated for every sample by estimate.^32^ The macrophage cell fractions were also calculated for each sample by CIBERSORT.^33^

### 2.7 The real and algorithmically evaluated immunotherapy data

Here, three datasets with samples treated with anti‐PD‐1 were also collected, including GC cohort,^34^ skin cutaneous melanoma cohort,^35^ and bladder cancer cohort (IMVigor210).^36^

Using the transcriptome of each bulk GC tissue sample, the Submap algorithm of GenePattern^37^ and Tumor Immune Dysfunction and Exclusion (TIDE) algorithm^38^ were performed to predict the immunotherapy response information for every GC sample (FDR <0.05).

### 2.8 Functional enrichment analysis

We performed Clusterprofiler to explore the functionally enriched Kyoto Encyclopaedia of Genes and Genome pathways and GSVA to explore the hallmark gene sets (MSigDB) associated with subgroups.

### 2.9 Identification of hub genes

Based on the protein–protein interactions from STRING, the top 50 genes were defined as hub genes (potential therapeutic targets) by the 12 algorithms (Degree, Edge Percolated Component, Maximum Neighborhood Component, Density of Maximum Neighborhood Component, Maximal Clique Centrality, Bottleneck, EcCentricity, Closeness, ClusteringCoefficient, Radality, Betweenness, and Stress) using the CytoHubba plugin in Cytoscape.^39^, ^40^

## 3 RESULTS

### 3.1 Limitations of the threshold‐based scoring models

**FIGURE 1:**
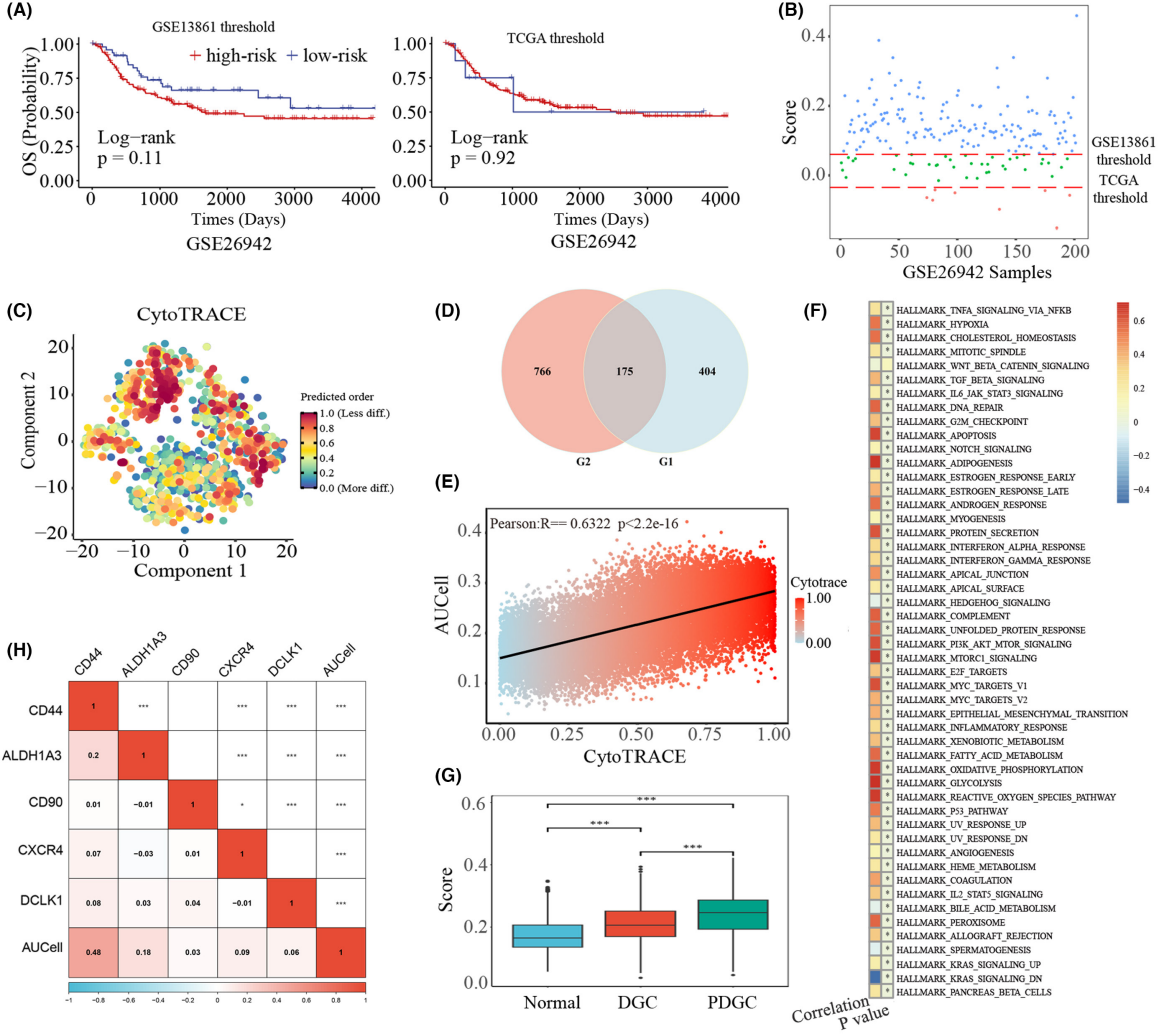
Identification of stemness‐related signature genes. (A) The survival outcomes of GSE26942 predicted by the thresholds of GSE13861 and The Cancer Genome Atlas (TCGA), respectively. (B) The samples of GSE26942 classified into high risk and low risk using the thresholds identified in GSE13861 and TCGA, respectively. The green dots denoted samples with the GSE13861 threshold classified as low‐risk, but high‐risk with the TCGA threshold. (C) Uniform manifold approximation and projection (UMAP) visualization of CytoTRACE scores of malignant cells in GSE134520. CytoTRACE is an algorithm to evaluate the stemness of single cells based on their expression profiles. (D) The overlapping genes between *G*_1_ and *G*_2_ were stemness‐related signature genes. *G*_1_ represented genes that were positively correlated with CytoTRACE scores, and *G*_2_ represented genes differentially upregulated in malignant cells. (E) The correlation between AUCell score of the signature genes and CytoTRACE score in HRA002108. (F) The correlation between AUCell score of the signature genes and 50 hallmark pathways in HRA002108. (G) The AUCell scores of normal samples, DGC (differentiated gastric cancer) samples, and PDGC (poorly differentiated GC) samples. Statistical significance: *p < 0.05, **p < 0.01, ***p < 0.001. (H) The correlation between the AUCell score of the signature genes and known GC stem cell marker genes.

In real clinical scenarios, a definite standard is needed to determine whether a given patient is at high risk or low risk to assist in clinical treatment decisions. However, the existing threshold‐based scoring models lack a definite threshold applicable to different GC samples. Here, we used the model proposed by Chen et al.^12^ as an example to illustrate the limitations of the threshold‐based scoring models. The threshold used by Chen et al. is the median values of the corresponding dataset. In GSE13861 and TCGA, survival outcomes are significantly different between the two subtypes using the stemness‐related scoring model proposed by Chen et al. (Figure S1A). However, when the two thresholds of GSE13861 and TCGA were applied in GSE26942, there was no significant difference in survival outcomes between the two groups classified by the two thresholds (Figure 1A). Moreover, 50 samples classified as high‐risk using the threshold of GSE13861 changed to low risk using the threshold of TCGA (Figure 1B). The similar result was also observed in the other datasets (Figure S1). Overall, the lack of a definite robustness threshold prevents these threshold‐based scoring models from being applied clinically.

### 3.2 Identification of stemness‐related signature genes using scRNA‐Seq data

The flowchart was shown in Figure S2. Here, we used GC scRNA‐Seq data, GSE134520, to identify stemness‐related signature genes. First, we calculated CytoTRACE scores of malignant cells to estimate their stemness (Figure 1C; Figure S3A). Second, 579 genes showed positive correlation with the stemness scores were set as *G*_1_ (Pearson *R* > 0.2 and FDR <0.05), and genes upregulated in malignant cells were set as *G*_2_. The 175 overlapping genes between *G*_1_ and *G*_2_ were defined as stemness‐related signature genes (Figure 1D; Table S1). An additional single‐cell RNA‐Seq data of GC, HRA002108, was further used to evaluate the identified signature genes (Figure S3B,C). As shown in Figure 1E,F, the AUCell scores of these genes were positively correlated with the CytoTRACE scores, as well as the activity scores of 50 hallmark pathways, such as HYPOXIA, P53_PATHWAY, TNFA_SIGNALING_VIA_NFKB and epithelial–mesenchymal transition (EMT). Moreover, based on the pathological grade of Wang et al. the AUCell scores of poorly differentiated GC samples were significantly higher than those of differentiated GC samples (Figure 1G). And the AUCell scores were positively correlated with the expression of known GC stem cell marker genes (Figure 1H), including *CD44*,^41^ *CD90*,^42^ *CXCR4*,^43^ *DCLK1*,^44^, ^45^ and *ALDH1A3*.^46^, ^47^ Especially for *CD44*, the correlation coefficient reached 0.48, and cells with larger enrichment scores had higher expressions of *CD44* (Figure S4). Overall, these results laterally confirmed the reliability of our identified stemness‐related signature genes.

### 3.3 Classify GC samples into two subtypes based on stemness‐related signature genes in training data

**FIGURE 2:**
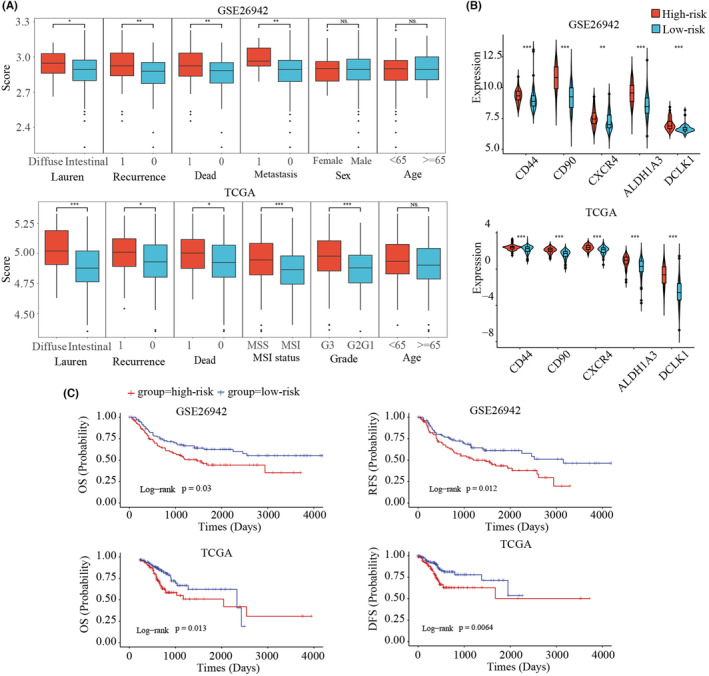
Analysis of the stemness‐related signature genes in two bulk tissue datasets. (A) The enrichment scores of 175 stemness‐related signature genes of the two groups classified by the corresponding clinical features. The group denoted as 1 (0) represented recurrence (non‐recurrence), dead (non‐dead), and metastasis (non‐metastasis) patients. MSI and MSS were microsatellite instability and microsatellite stability patients, respectively. Grades 1, 2, and 3 were G1, G2, and G3, respectively. The *p*‐value was calculated using Wilcoxon rank‐sum test. Statistical significance: *p < 0.05, **p < 0.01, and ***p < 0.001. (B) The expression of known gastric cancer stem cell marker genes in high‐risk and low‐risk groups. (C) The survival analysis for two subtypes identified in TCGA and GSE26942, respectively.

Using GC samples from TCGA and GSE26942 as training data, the ssGSEA algorithm was used to quantitatively measure the enrichment scores of the signature genes of each sample. Based on the clinical information, we found that the diffuse subtype had significantly higher enrichment scores than that of the intestinal subtype (Figure 2A). Previous studies have shown that enteric type has a higher degree of differentiation and a better prognosis, while diffuse type has a lower degree of differentiation and a worse prognosis, which is consistent with our results. Similarly, the death group, the recurrence group, the Grade 3 group and the distant metastasis group also had higher enrichment scores (Figure 2A). In addition, the enrichment score was not affected by age or sex. Using the median value of the enrichment scores as the threshold to classify the samples into two subtypes, the result showed that maker genes of GC stem cells had significantly higher expression in the high enrichment score (high risk) group (Figure 2B). Further, the high‐risk group had significantly shorter OS and RFS/DFS (Figure 2C). These results were consistent with our consensus, namely, the group with higher stemness (high risk) had poorer prognosis. Overall, these results suggested that our developed stemness‐related signature genes could accurately classify GC samples into two subtypes with different degree of stemness and prognosis.

### 3.4 Molecular characteristics and biological functions of GC subtypes

**FIGURE 3:**
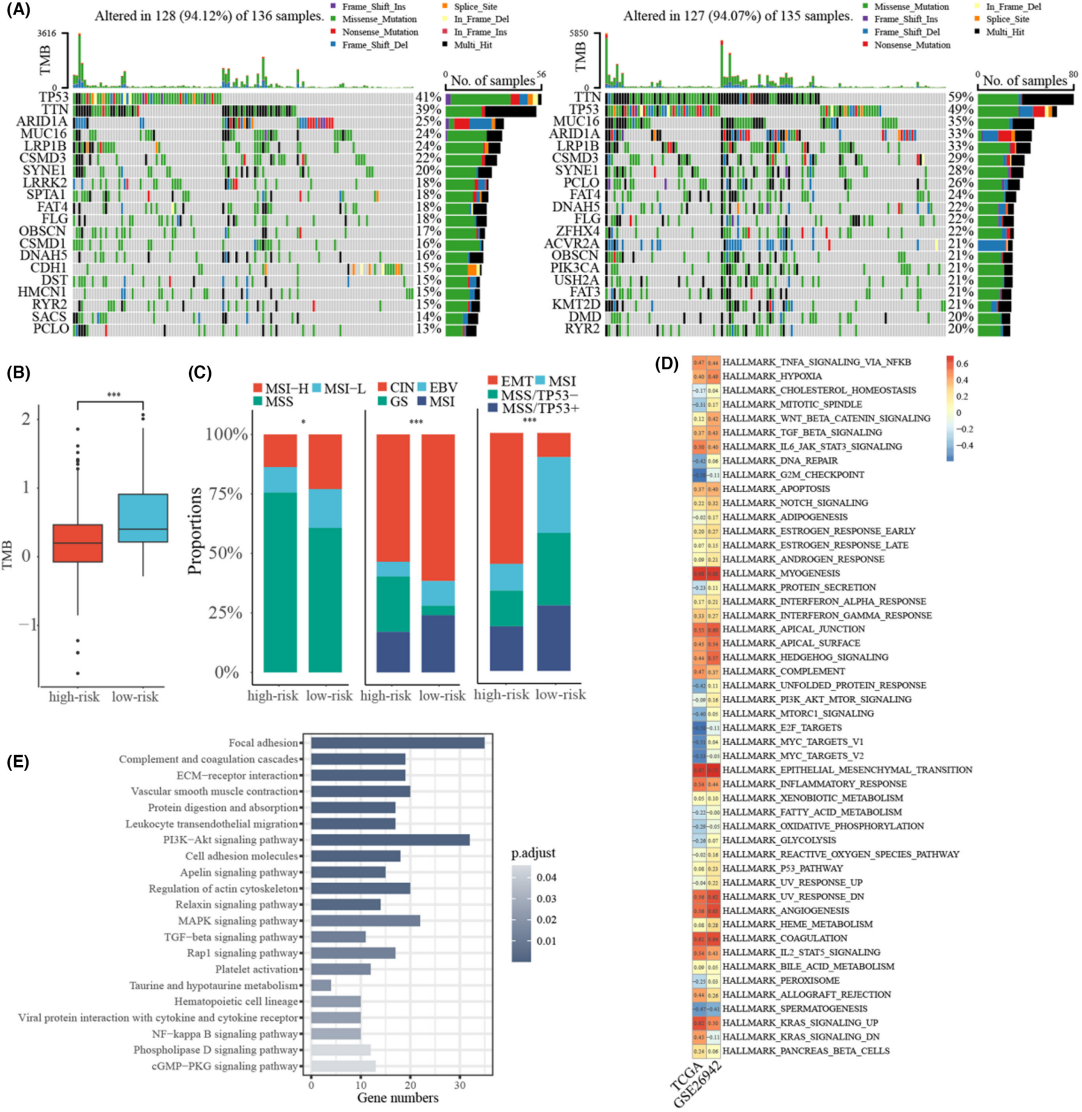
Genomic characterization and functional analysis of two subtypes. (A) Somatic mutation landscape of high‐risk group and low‐risk group in The Cancer Genome Atlas (TCGA). (B) Comparison of tumor mutation burden between high‐risk and low‐risk samples in TCGA. (C) The proportions of MSI status, TCGA, and Asian Cancer Research Group subtypes in high‐risk and low‐risk groups, respectively. (D) Correlation between the enrichment scores of the signature genes and hallmark pathways. (E) Functional enrichment analysis of uniformly upregulated genes in both TCGA and GSE26942 high‐risk group. Statistical significance: *p < 0.05, **p < 0.01, and ***p < 0.001.

First, somatic mutations in two subtypes in the TCGA cohort were investigated. Most of the highly frequent mutation genes were shared in both two subtypes, such as *TP53*, *TTN*, and *MUC16* (Figure 3A). Specifically, *CDH1* mutation was only frequently observed in the high‐risk group. It has been reported that mutations in *CDH1* are associated with GC, and its loss of function may contribute to cancer progression by increasing proliferation, invasion, and/or metastasis.^48^ Further analysis showed that tumor mutation burden (TMB) levels were significantly higher in the low‐risk group than those in the high‐risk group (Figure 3B). TMB reflects the repair of DNA damage in cancer cells and is closely related to microsatellite instability (MSI). Also, we found that the proportion of microsatellite stability (MSS) in the high‐risk group was significantly higher than that in the low‐risk group (Figure 3C). In addition, we analyzed two landmark GC molecular models, including TCGA^49^ and ACRG^50^ molecular classifications, in two subtypes. The TCGA molecular classification mainly includes Epstein–Barr virus, MSI, genomically stable tumors, and chromosomal instability. The proportion of genomically stable tumors subtypes in high‐risk group was significantly higher than that in low‐risk group, while the proportion of MSI and Epstein–Barr virus was lower than that in low‐risk group (Figure 3C). For the ACRG molecular classification, mainly including MSS/TP53+, MSS/TP53+, MSI, and MSS/EMT, we found a significantly higher proportion of EMT subtype and a significantly lower proportion of MSI subtype in the high‐risk group (Figure 3C). Previous studies have shown that the genomically stable tumors subtype and EMT subtype correspond to a worse prognosis,^51^ which was consistent with our results.

Next, we further analyzed the functions of two subtypes. The GSVA analysis was performed on 50 cancer hallmark pathways in two risk groups in the two training cohorts. Consistent with the above result of single‐cell RNA‐Seq, we found that cancer‐related pathways such as EMT, KRAS_SIGNALING_UP, and ANGIOGENESIS were significantly positively correlated with enrichment scores (Figure 3D). Using Wilcoxon rank‐sum test with FDR <5%, 1921 and 1065 DEGs were detected between the high‐risk and low‐risk groups for TCGA and GSE26942, respectively. The two lists of DEGs shared 673 genes and the concordance score was 100%, which was unlikely to happen by chance (binomial test, all *p* < 2.20E‐16). Furthermore, common upregulated genes in high‐risk group were significantly enriched in tumor metastasis and stemness associated pathways, such as focal adhesion, ECM‐receptor interaction, cell adhesion molecules, TGF‐β signaling pathway, MAPK signaling pathway, and PI3K‐Akt signaling pathway (Figure 3E).

### 3.5 Develop an individualized stemness‐related signature to predict prognosis

**FIGURE 4:**
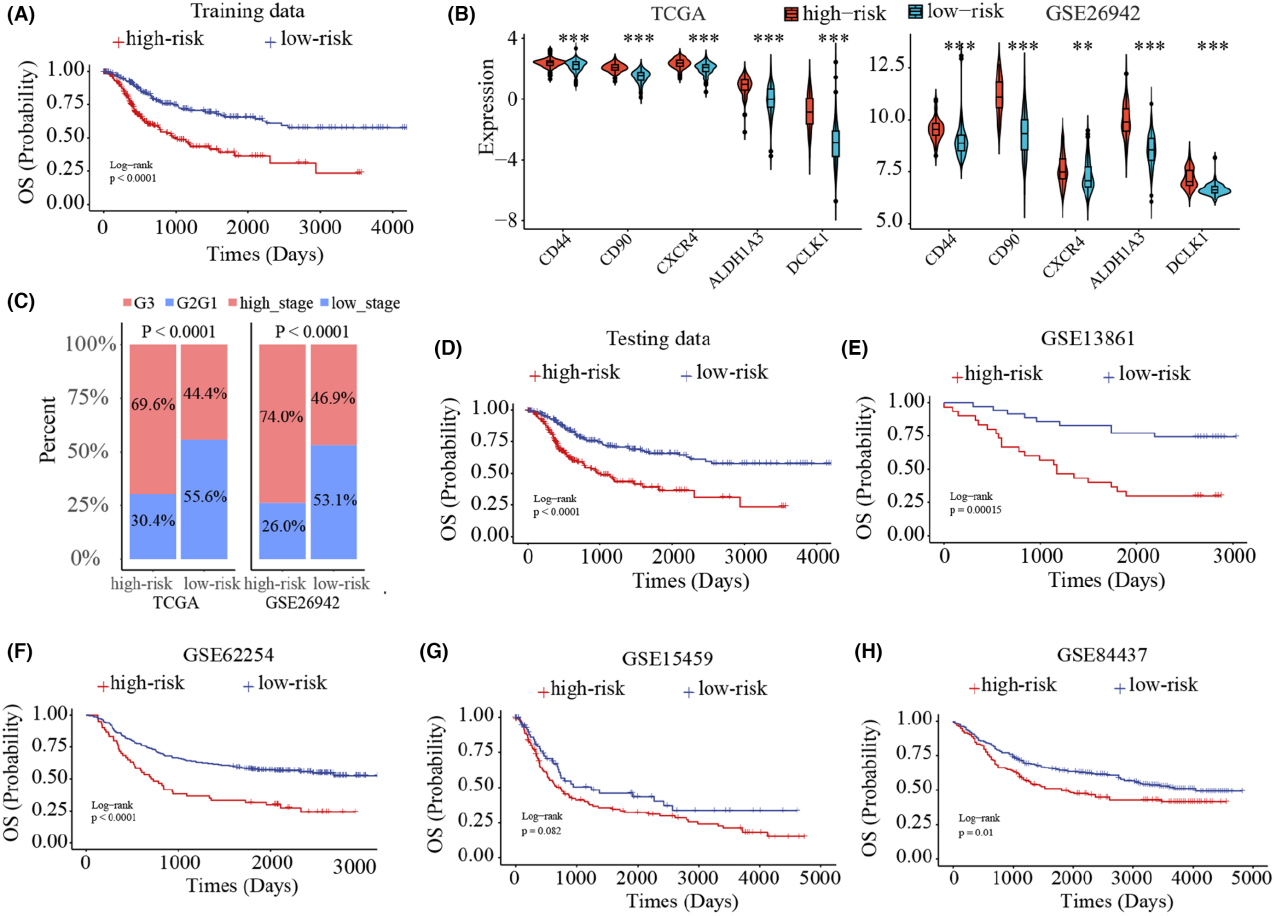
Construction and evaluation of 47‐gene pairs (47‐GPS). (A) The Kaplan–Meier analysis of samples in training data, namely the merge data of The Cancer Genome Atlas (TCGA) and GSE26942. (B) The expression of gastric cancer stem cell marker genes in two groups classified by 47‐GPS. (C) The percents of high‐risk and low‐risk patients in different grades (TCGA) and stages (GSE26942). The III and VI stage patients were defined as high_stage, and the I and II stage patients were low_stage. The Kaplan–Meier analysis of samples in testing data (D), GSE13861 (E), GSE62254 (F), GSE125459 (G), and GSE84437 (H).

In real clinical scenarios, a signature that can predict prognosis of GC patients one by one is important to guide clinical treatment. The REOs of gene pairs has been reported to be robust to sample composition and measurement platforms, and has been applied to develop individually diagnostic and prognostic signature.^21^ Thus, we tried to develop a stemness‐related REO signature that can predict prognosis of GC patients one by one based on the above classified high‐risk and low‐risk subtypes in TCGA and GSE26942. First, we respectively identified 568,289 gene pairs and 446,388 gene pairs with reversal REOs between high‐risk and low‐risk groups in TCGA and GSE26942. There were 7534 overlapped gene pairs for the two datasets, and 99.48% (7495) of them had the same reversal REO patterns (binomial test, *p* < 2.20E‐16). Among the 7495 gene pairs, we further obtained 3212 gene pairs whose REOs were significantly associated with patient OS in the integrated data with 270 samples from TCGA and GSE26942 (FDR <0.05, univariate Cox model). These gene pairs were defined as candidate prognostic gene pairs. Then, using the forward selection procedure described in Methods section, we obtained an optimal set of 47‐gene pairs (47‐GPS), denoted as 47‐GPS, with the largest C‐index value (74%) based on the half voting rule (Table S2). The two groups classified by 47‐GPS had significantly different OS not only in the integrated data (Figure 4A), but also in TCGA and GSE26942, respectively (Figure S5A). The similar results were also observed when clinical factors were adjusted (Figure S5B). Moreover, the high‐risk group showed higher expression of the known maker genes of GC stem cells (Figure 4B; Figure S6), and had a lower degree of differentiation (Figure 4C).

### 3.6 Validation of the 47‐GPS in testing datasets

The performance of the 47‐GPS was validated in four independent testing datasets measured by different laboratories and platforms (GSE13861, GSE62254, GSE15459, and GSE84437). For the pooled data from the four datasets, the high‐risk patients had significantly worse survival outcomes than low‐risk patients (Figure 4D). For GSE13861 measured by GPL6884, 65 samples, with RFS information and OS information, were classified as high‐risk (30) and low‐risk (35) groups, and the two groups showed significantly different OS (Figure 4E) and RFS (Figure S7A). For GSE62254 and GSE15459 measured by GPL570, different OS (Figure 4F,G) and DFS (Figure S7B) were also observed between two groups. The similar result was also observed in GSE84437 measured by GPL6947 (Figure 4H). The 47‐GPS was also significantly related with OS even after adjustment for clinical factors, including sex, age, grade, and M‐stage (Figure S8).

Overall, the 47‐GPS could accurately predict prognosis outcomes even for GC patients profiled by different platforms and laboratories.

### 3.7 The relationship between the 47‐GPS and immunotherapy

**FIGURE 5:**
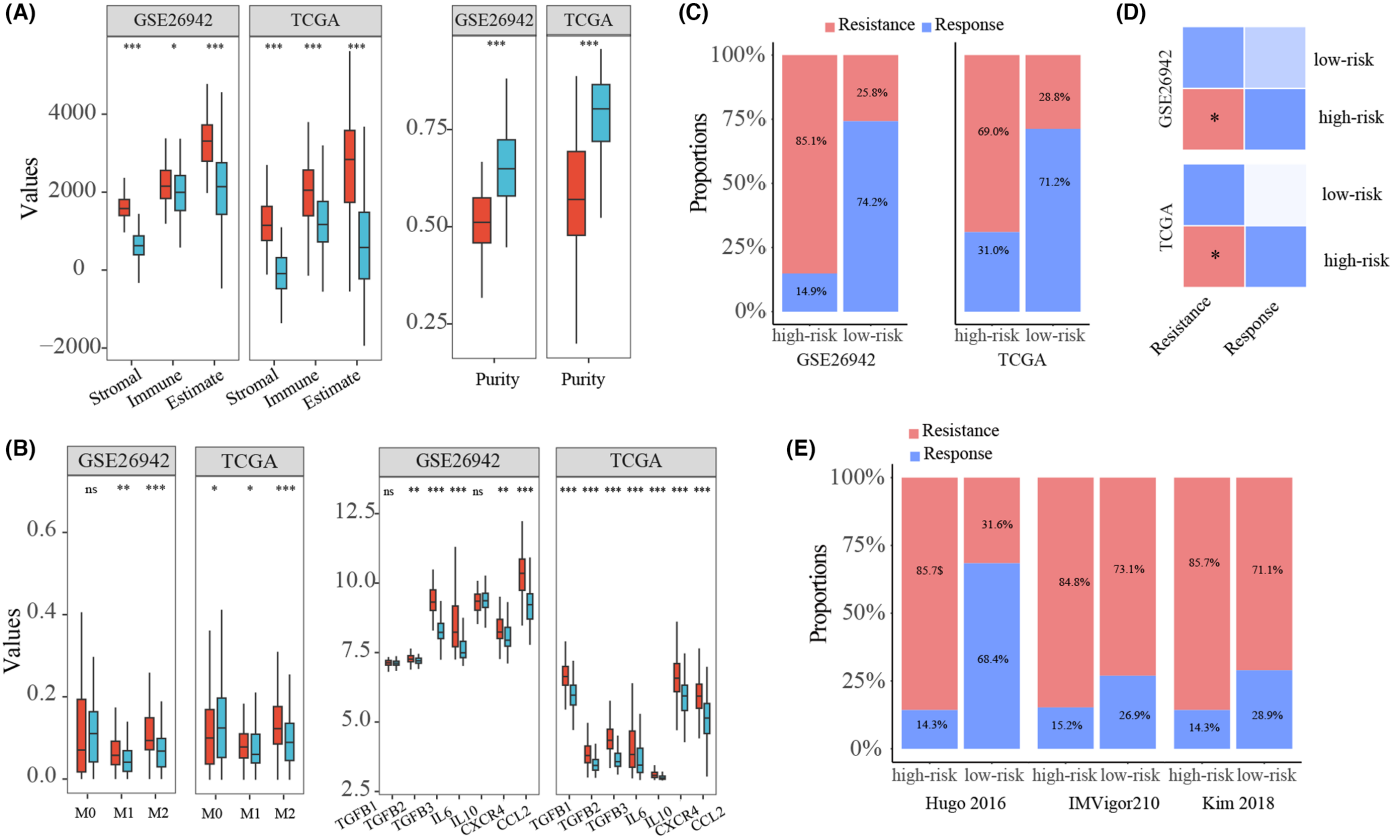
The performance of 47‐gene pairs in predicting immunotherapy outcomes. (A) Comparison of the immune score, stromal score, ESTIMATE score and tumor purity between the high‐risk group and low‐risk group in GSE26942 and TCGA, respectively. (B) Comparison of infiltration densities of macrophage and expression of M2 polarization factor between the high‐risk group and low‐risk group. (C) TIDE analysis for high‐risk and low‐risk samples. (D) Submap analysis for high‐risk and low‐risk samples. (E) The proportions of response samples and resistance samples in high‐risk group and low‐risk group, respectively, for the real immunotherapy data. Hugo 2016, IMVigor210, and Kim 2018 represented GC cohort, skin cutaneous melanoma cohort, and bladder cancer cohort, respectively.

Based on ESTIMATE, the immune score and stromal score of the sample were calculated. We found that the high‐risk group had significantly higher immune scores and stroma scores, and significantly lower tumor purity, compared with the low‐risk group (Figure 5A; Figure S9A). Furthermore, the high‐risk group was significantly associated with a high abundance of M2 macrophages (Figure 5B; Figure S9B) using CIBERSORT analysis. Meanwhile, we observed M2 polarization regulators were highly expressed in high‐risk group (Figure 5B; Figure S9B). These results were consistent with current reports that higher M2 cell abundance may lead to poorer outcomes. Taken together, the high‐risk group patients might be less sensitive to immunotherapy owing to the stromal and M2 macrophage‐mediated immunosuppression effects.^16^

Then, the immunotherapy responses of the high‐risk and low‐risk groups were assessed via TIDE algorithm. Compared with the low‐risk group, the proportion of immune resistance in the high‐risk group was significantly higher than that in the low‐risk group, indicating the resistance of the high‐risk group to immunotherapy (Figure 5C; Figure S9C). Simultaneously, we also used submap analysis to assess anti‐PD‐1 immunotherapy responses between high‐risk and low‐risk groups. The results demonstrated that the high‐risk patients had no response to anti‐PD‐1 immunotherapy (Figure 5D; Figure S9D). Consistent with the above prediction, among the three sets of real immunotherapy response data, the proportion of immunotherapy resistance patients was higher in the high‐risk group than that in the low‐risk group (Figure 5E).

### 3.8 Identification of potential therapeutic targets and drugs for high‐risk group

**FIGURE 6:**
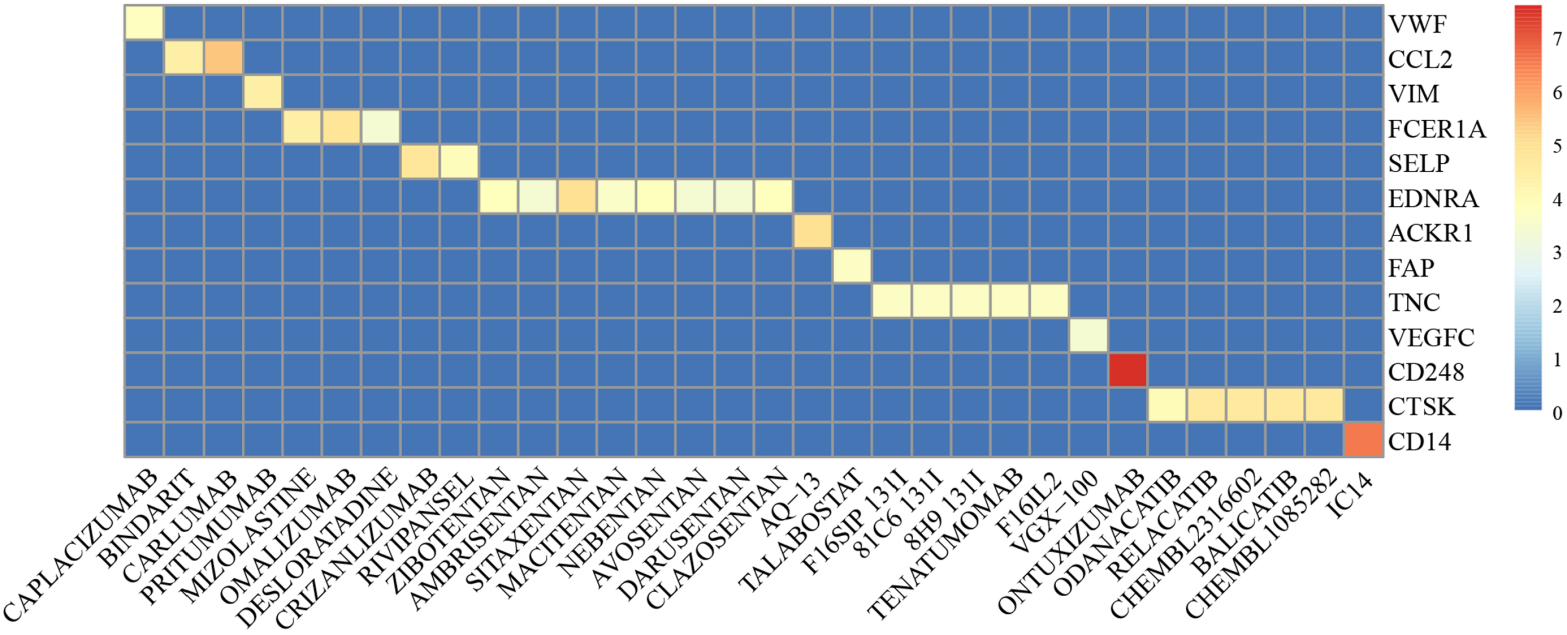
Identification of potential therapeutic targets and drugs.

With FDR <0.05, DEGs between the high‐risk and low‐risk samples were identified in six datasets (GSE26942, TCGA, GSE15459, GSE62254, GSE13861, and GSE84437), respectively. The concordance scores of any two lists of DEGs are above 99% (Table S3), indicating that the stable reproducible differential expression signals could be detected between our classified high‐risk and low‐risk groups. Uniformly upregulated genes in all six datasets were further screened, totaling 359 genes. These genes were significantly enriched in cancer‐related pathways, such as focal adhesion, ECM‐receptor interaction, PI3K‐Akt signaling pathway, and cell adhesion molecular pathway (Figure S10). Based on the protein–protein interactions from the STRING database, we obtained a sub‐network involved in the 359 uniformly upregulated genes, consisting of 322 nodes and 2113 edges. Twelve algorithms were applied to identify the top 50 hub genes by Cytohubba plugin in the gene interaction network. Furthermore, we merged hub genes identified by 12 methods, and finally obtained 150 genes. Among them, 13 genes targeted by 32 potential drugs were identified using the Drug‐Gene Interaction of gene‐drug interaction data (Figure 6). For example, *CD248* (Endosialin) has been reported to be involved in tumor cell vascular adhesion and migration, neoangiogenesis, local invasion, and metastasis.^52^ Ontuxizumab, a class of monoclonal antibodies, targeted *CD248*, which interferes with the function of endosialic acid.^53^ It has been reported that treatment with ontuxizumab shrink gastrointestinal stromal tumors.^54^

## 4 DISCUSSION

We identified 175 stemness‐related genes, and based on the enrichment scores of these genes using ssGSEA, the samples in training data were grouped into two subtypes. The known GC stemness marker genes, such as *CD44* and *ALDH1A3*, were differentially expressed in these two subtypes, and the two subtypes demonstrated different genomic characteristics and survival times. Using the subtypes as criteria, the 47‐GPS signature was developed based on the within‐sample REOs of gene pairs. The prognostic efficacy of 47‐GPS could be validated in four independent datasets detected by different laboratories and platforms. To evaluate the performance of 47‐GPS in pan‐cancer, we collected another 21 cancer types, each including at least 150 samples with survival information. Except for BLCA (bladder cancer) and KIRP (kidney renal papillary cell carcinoma), the high‐risk samples classified by the 47‐GPS do not show significantly worse outcomes in the other 19 cancer types (Figure S11), indicating that the 47‐GPS is not a common prognostic signature for pan‐cancer.

Our study results have demonstrated that the high‐risk group predicted by the 47‐GPS exhibits higher stemness and is associated with poorer prognosis. To further validate this, we analyzed the enrichment scores of five pathways (WNT‐β, TGF‐β, NOTCH, EMT, and HEDGEHOG), closely associated with tumor stemness, metastasis, and recurrence. The result showed that the high‐risk group exhibited significantly higher enrichment scores compared to the low‐risk group (Figure S12). Compared with low‐risk samples, high‐risk samples also had lower TMB levels and higher MSS sample proportion, M2 cell proportion and M2 polarization factor expression, which was consistent with previous reports. It has been reported that cancer stem cells recruit tumor‐associated macrophages and affect the biological state of macrophages.^55^ Macrophages mainly include antitumor (M1) phenotype and pro‐tumor (M2) phenotype. Cancer stem cells could secrete a variety of factors that promote the polarization of macrophages, thereby inducing the evolution of macrophages into pro‐tumor phenotype.^56^ This may cause immunosuppression and lead to resistance to checkpoint inhibitor therapy.^57^ Many evidences have shown the correlation between stemness and immune evasion.^58^ Consistent with these reports, we also found that the high‐risk group with higher stemness was significantly associated with immunotherapy resistance. For high‐risk patients, we further identified a number of potential therapeutic targets and corresponding drugs. For example, *EDNRA* is the receptor for endothelin‐1, which can regulate the proliferation and invasion of GC cells.^59^ Macitentan, a drug targeting *EDNRA*, has been reported in multiple studies, which can improve tumor immunotherapy outcomes and inhibit tumor metastasis.^60^ Tenascin‐c (*TNC*) is an extracellular matrix protein that belongs to the Tenascin gene family. Its expression is closely related to the initiation and metastasis of GC.^61^ In addition, knockout of *TNC* expression can inhibit the proliferation, migration and invasion of GC cells in vitro by inducing cell cycle arrest in G0/G1 phase, and inhibit peritoneal metastasis in vivo.^61^ Overall, the identified therapeutic targets and drugs provide candidate treatments for high‐risk GC patients, which deserve for further study.

Comparing with the previous threshold‐based scoring models, the 47‐GPS could accurately classify a given sample into high risk or low risk, providing an auxiliary tool in clinical GC treatment. However, this study also has certain limitations. First, some subtle quantitative gene expression information may be lost in the rank‐based signature,^22^ 47‐GPS. Second, due to the high sparsity of single‐cell transcriptomes, we cannot verify the efficacy of 47‐GPS at the single‐cell level. Third, the GC samples treated with immunotherapy are limited, and a prospective study with more samples is needed to further verify the performance of 47‐GPS in predicting immunotherapy responses.

Among 72 genes, consisting of 47‐GPS, 19 genes were differentially upregulated in high‐risk group, and most of them were associated with tumor stemness, and tumor initiation and metastasis (Table S4). For example, gene *MGP*, a member of the osteocalcin/matrix Gla protein family, has been reported to drive stemness and tumor initiation in ovarian cancer.^62^ Except for *MGP*, *NDN*,^63^ *SERP2*,^64^ *FSTL1*,^65^ and *CDH5* ^66^ were also closely related to tumor stemness.

## 5 CONCLUSION

Overall, by integrating single‐cell RNA‐Seq data and bulk tissue data, we developed a qualitative stemness‐related signature, 47‐GPS, which can accurately predict patients' prognosis and immunotherapy responses at an individual level. This makes it applicable to real clinical scenarios and assists doctors in formulating appropriate treatment plans for GC patients.

## AUTHOR CONTRIBUTIONS

**Linyong Zheng:** Data curation (equal); formal analysis (equal); investigation (equal); methodology (equal); software (equal); supervision (equal); validation (equal); visualization (equal); writing – original draft (equal); writing – review and editing (equal). **Jingyan Chen:** Methodology (equal); validation (equal); visualization (equal). **Wenhai Ye:** Data curation (equal); methodology (equal); visualization (equal). **Qi Fan:** Data curation (equal); investigation (equal); methodology (equal); visualization (equal). **Haifeng Chen:** Investigation (equal); methodology (equal); writing – original draft (equal); writing – review and editing (equal). **Haidan Yan:** Funding acquisition (lead); writing – original draft (equal); writing – review and editing (equal).

## FUNDING INFORMATION

This work was supported by the Fujian Natural Science Foundation (Grant Number 2023J01755) and Fujian Provincial Clinical Medical Research Center for First Aid and Rehabilitation in Orthopaedic Trauma (Grant Number 2020Y2014).

## CONFLICT OF INTEREST STATEMENT

The authors declare that they have no conflict of interest.

## ETHICS STATEMENT

Ethics approval statement is not applicable to this article.

## PATIENT CONSENT FOR PUBLICATION

The section is not relevant to my manuscript, so it is “not applicable” for this section.

## Supporting information


Data S1.

## Data Availability

Data sharing is not applicable to this article as no new data were generated or analyzed in this study.
