# Supplementary material for: An individualized stemness‐related signature to predict prognosis and immunotherapy responses for gastric cancer using single‐cell and bulk tissue transcriptomes
Source: Cancer Med. 2024 Jan 3;13(1):e6908. doi: 10.1002/cam4.6908 (PMC10807574; doi:10.1002/cam4.6908)
Supplement: Supplementary file 1 — Data S1. [file CAM4-13-e6908-s001.docx]

**Supplemental Data**

**An individualized stemness-related signature to predict prognosis and immunotherapy responses for gastric cancer using single-cell and bulk tissue transcriptomes**

**Linyong Zheng^1^, Jingyan Chen^1^, Wenhai Ye^1^, Qi Fan^1^, Haifeng Chen^2^*, Haidan Yan^1,3^***

^1^Department of Bioinformatics, Fujian Key Laboratory of Medical Bioinformatics, School of Medical Technology and Engineering, Fujian Medical University, Fuzhou, 350122, China;

^2^Department of Gastrointestinal Surgery, Fuzhou Second Hospital, 350007, China;

^3^Key Laboratory of Ministry of Education for Gastrointestinal Cancer, the School of Basic Medical Sciences, Fujian Medical University, Fuzhou, 350122, China.

**Email addresses:**

^Linyong Zheng, Email:^ [^773844043@fjmu.edu.cn^](mailto:773844043@fjmu.edu.cn)

^Haidan Yan, Email:^ [^haidan1115@fjmu.edu.cn^](mailto:haidan1115@fjmu.edu.cn)

^Jingyan Chen, Email:^ [^cjy20021206@163.com^](mailto:cjy20021206@163.com)

^Wenhai Ye, Email:^ [^yewenhai11@163.com^](mailto:yewenhai11@163.com)

^Qi Fan, Email:^ [^funmoy@163.com^](mailto:funmoy@163.com)

^Haidan Yan, Email:^ [^haidan1115@fjmu.edu.cn^](mailto:haidan1115@fjmu.edu.cn)

^Haifeng Chen, Email:^ [^feng278990983@163.com^](mailto:feng278990983@163.com)

*Corresponding authors:

^Haidan Yan, Email:^ [^haidan1115@fjmu.edu.cn^](mailto:haidan1115@fjmu.edu.cn)

^Haifeng Chen, Email:^ [^feng278990983@163.com^](mailto:feng278990983@163.com)

**Supplemental Figures**

**
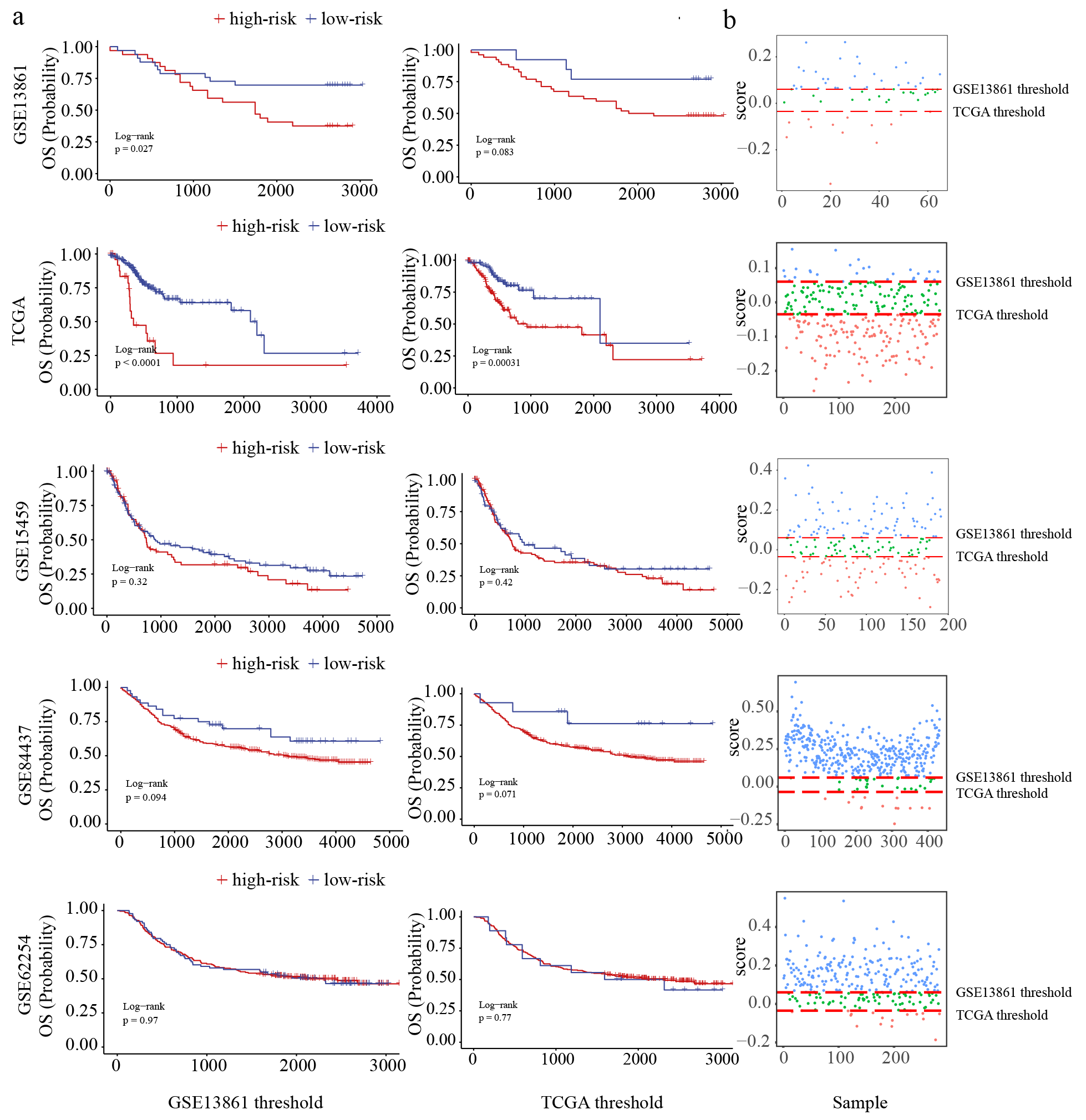
**

**Supplemental Fig. S1. Evaluate the performance of the GSE13861 threshold and TCGA threshold in different GC datasets.** **a** The survival outcomes for the two groups classified by the GSE13861 threshold and TCGA threshold, respectively. **b** The samples classified by the two thresholds. The green dots denoted samples with the GSE13861 threshold classified as low-risk, but high-risk with the TCGA threshold.

**
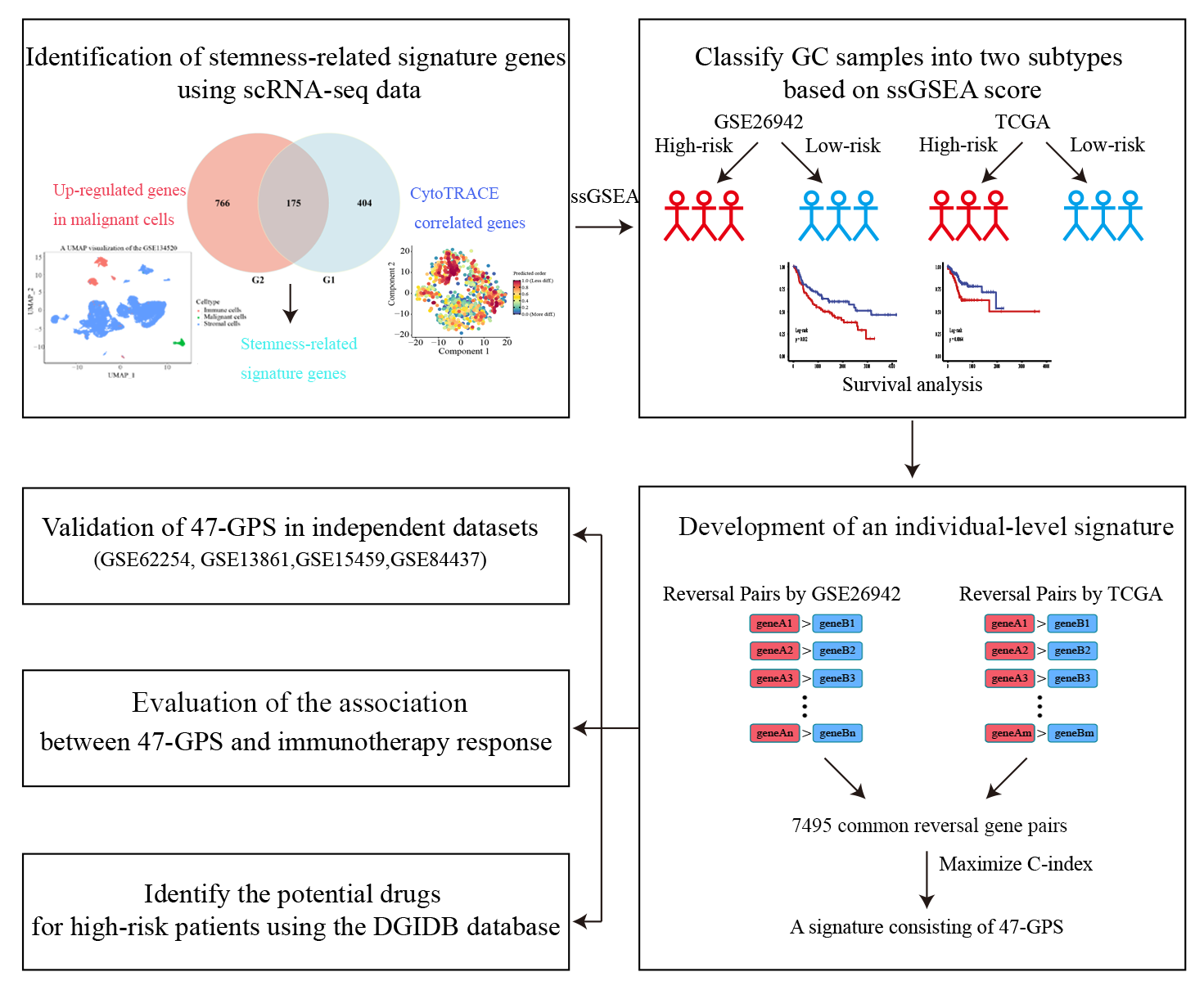
**

**Supplementary Fig. S2. The flowchart of this study.**

**
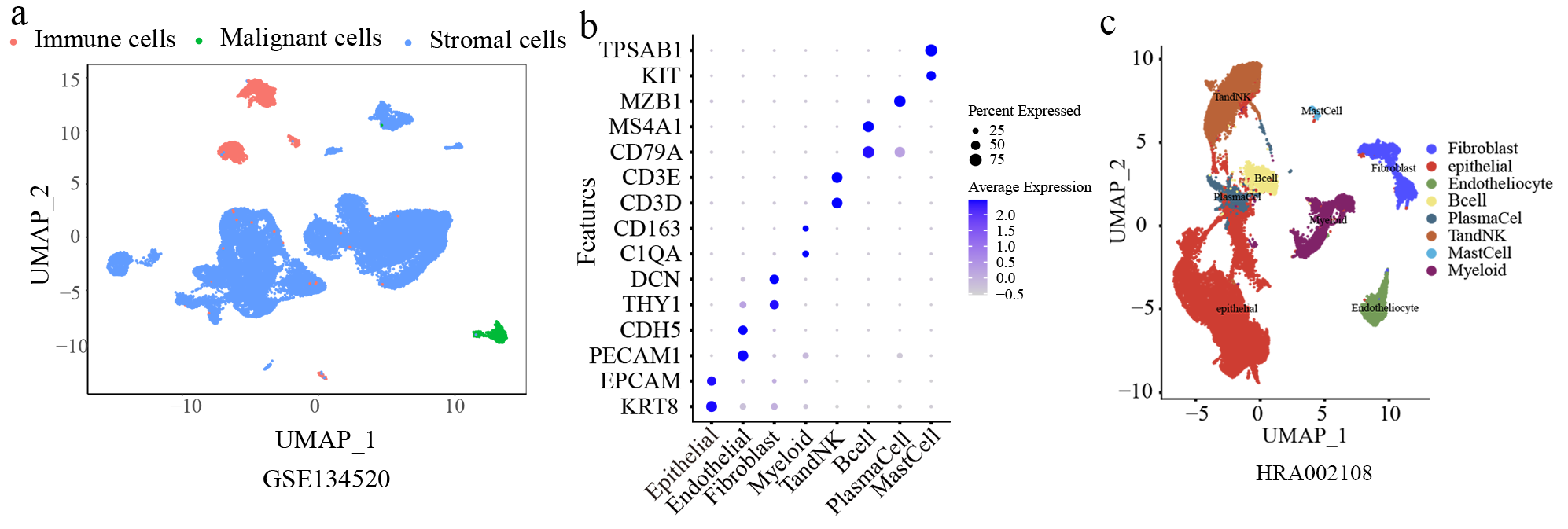
Supplemental Fig. S3. The cell type annotation of scRNA-seq data. a** Uniform manifold approximation and projection (UMAP) visualization of cells in GSE134520. **b** The expression of marker genes for different cell types. **C** UMAP plot of different cell types in HRA002108.

**
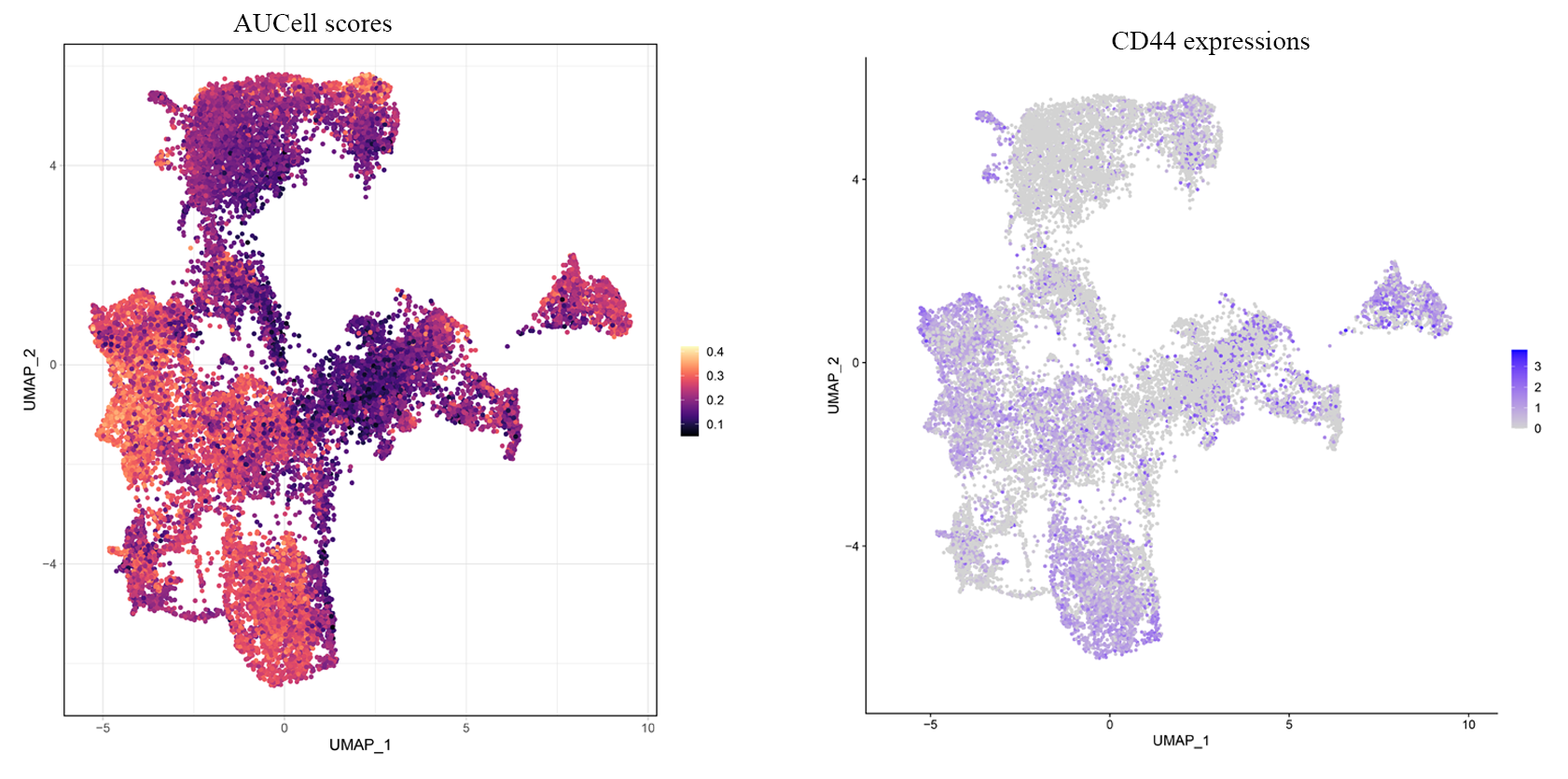
**

**Supplemental Fig. S4. The** **enrichment scores of signature genes and the expression of CD44 in tumor cells of HRA002108.**

**
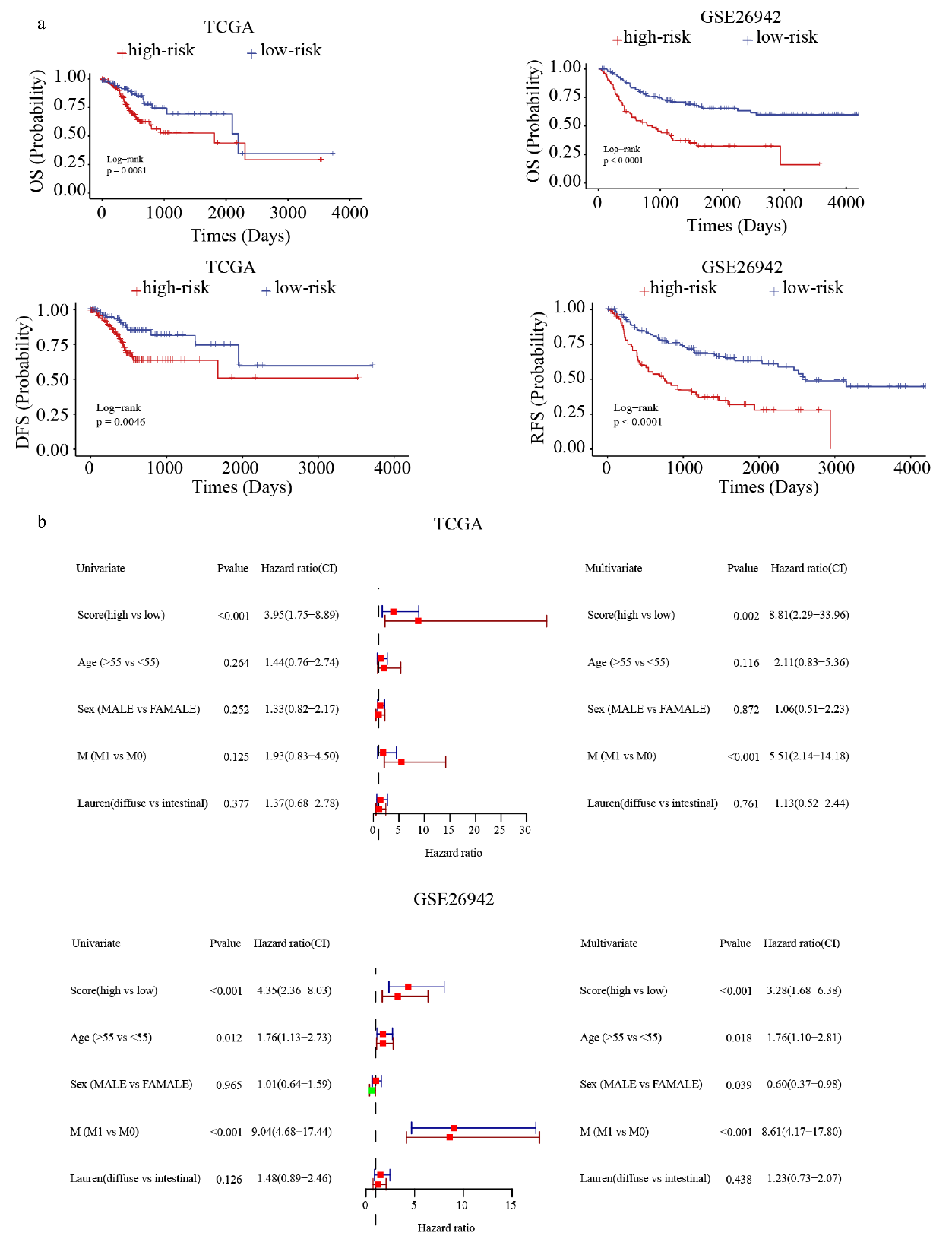
**

**Supplemental Fig. S5. The performance of 47-GPS in training data. a** The Kaplan–Meier analysis of samples in TCGA and GSE26942, respectively. **b** Cox Proportional Hazards model analysis of TCGA and GSE26942, respectively.

**
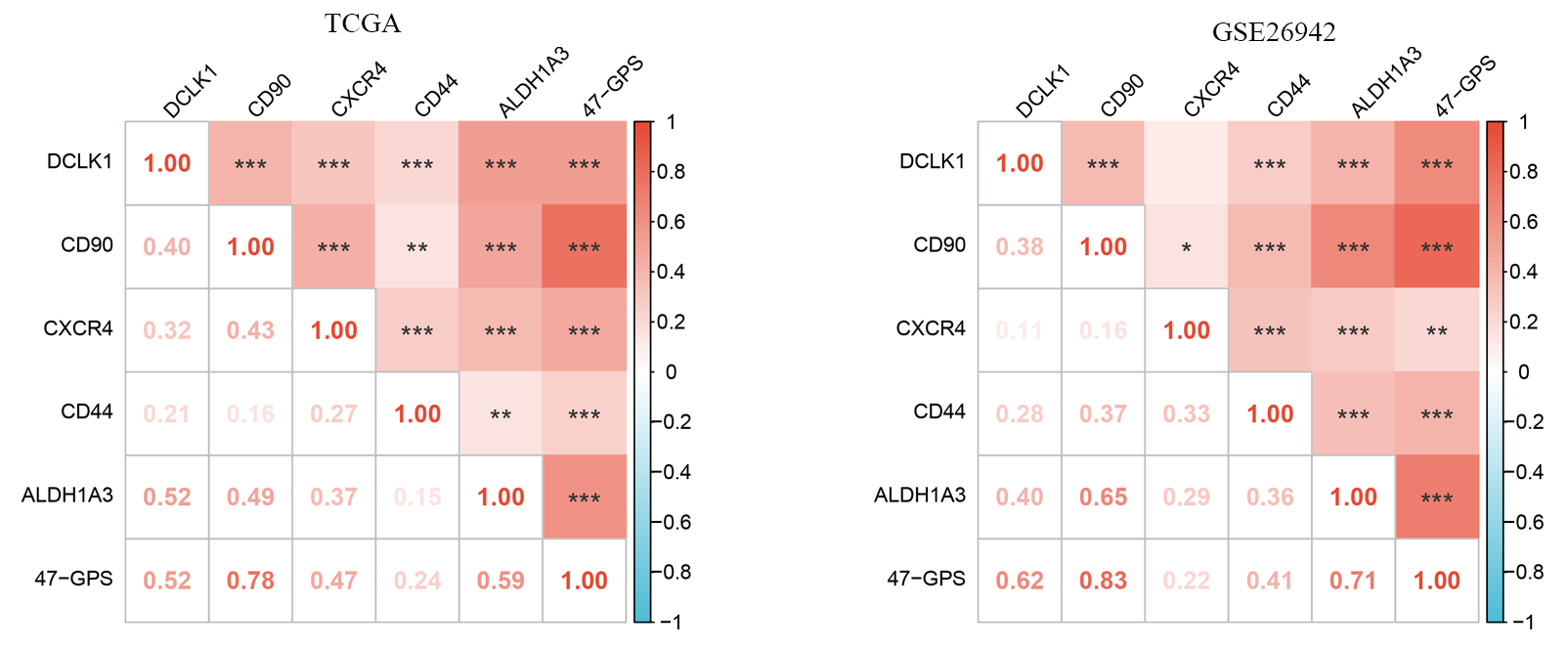
**

**Supplemental Fig. S6. The correlation between the 47-GPS value and the expression of known GC stem cell marker genes.** Among the 47 gene pairs of the 47-GPS, let *m* denote the number of gene pairs supported for high-risk in a GC patient, then the 47-GPS value is *m/47*. The larger 47-GPS value, the higher degree of stemness.

**
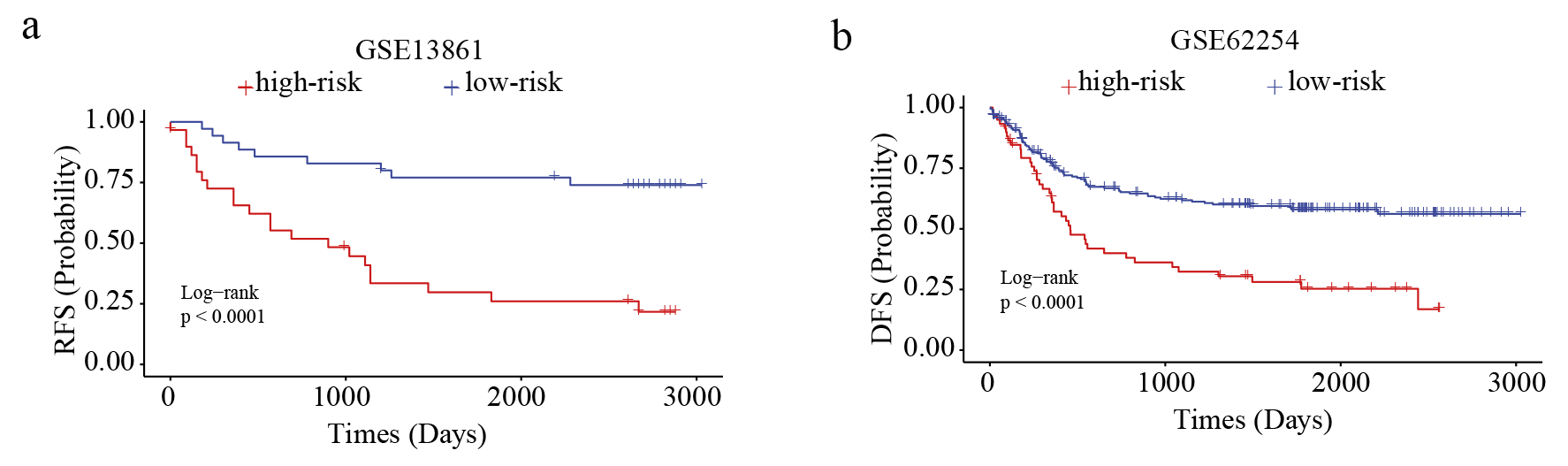
**

**Supplemental Fig. S7. The performance of 47-GPS in independent testing data.** The Kaplan–Meier analysis of samples in GSE13861 (**a**) and GSE62254 (**b**), respectively.


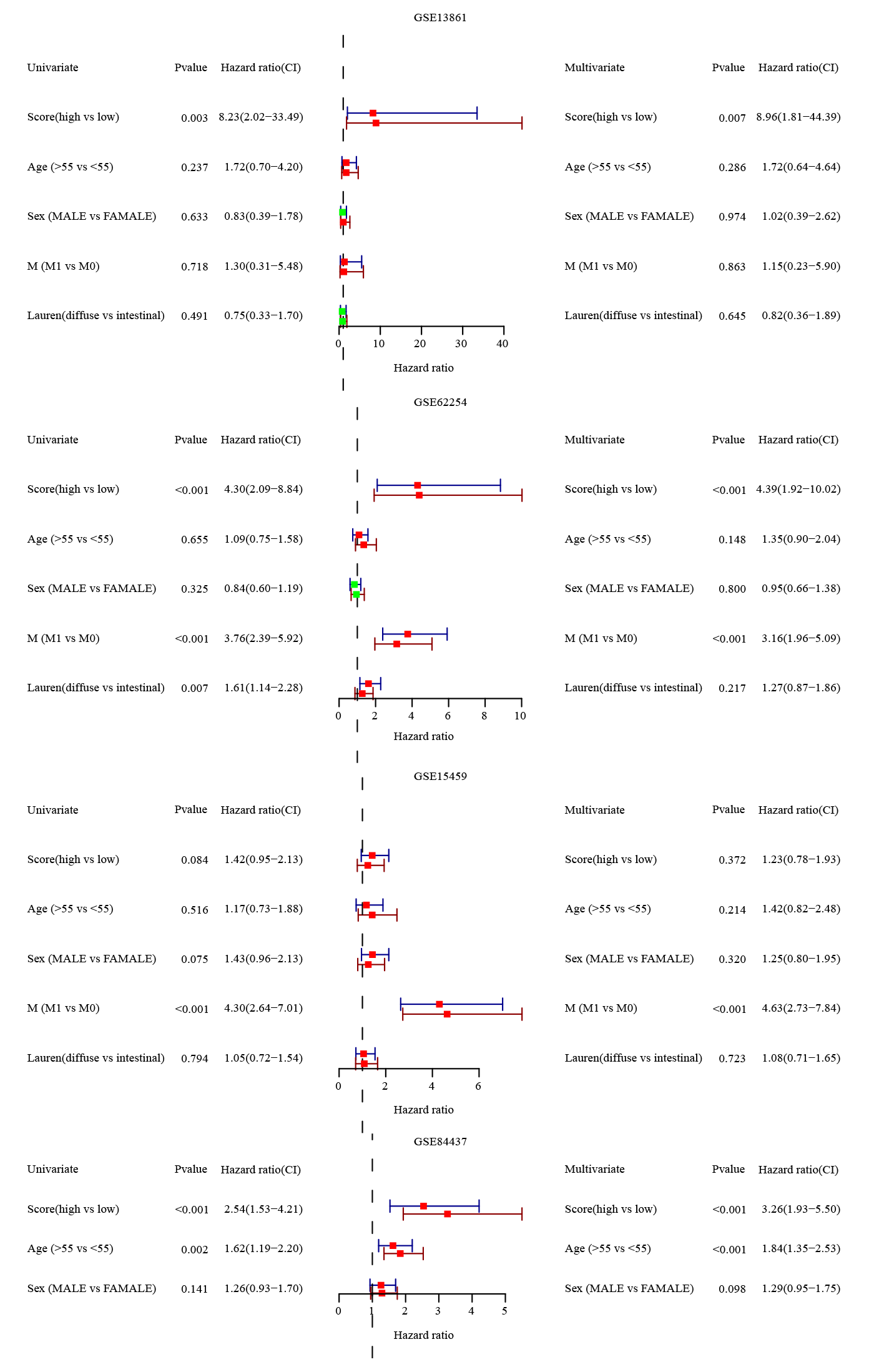


**Supplemental Fig. S8. The Cox Proportional Hazards Model analysis of samples in GSE13861, GSE62254, GSE15459 and GSE84437, respectively.**

**
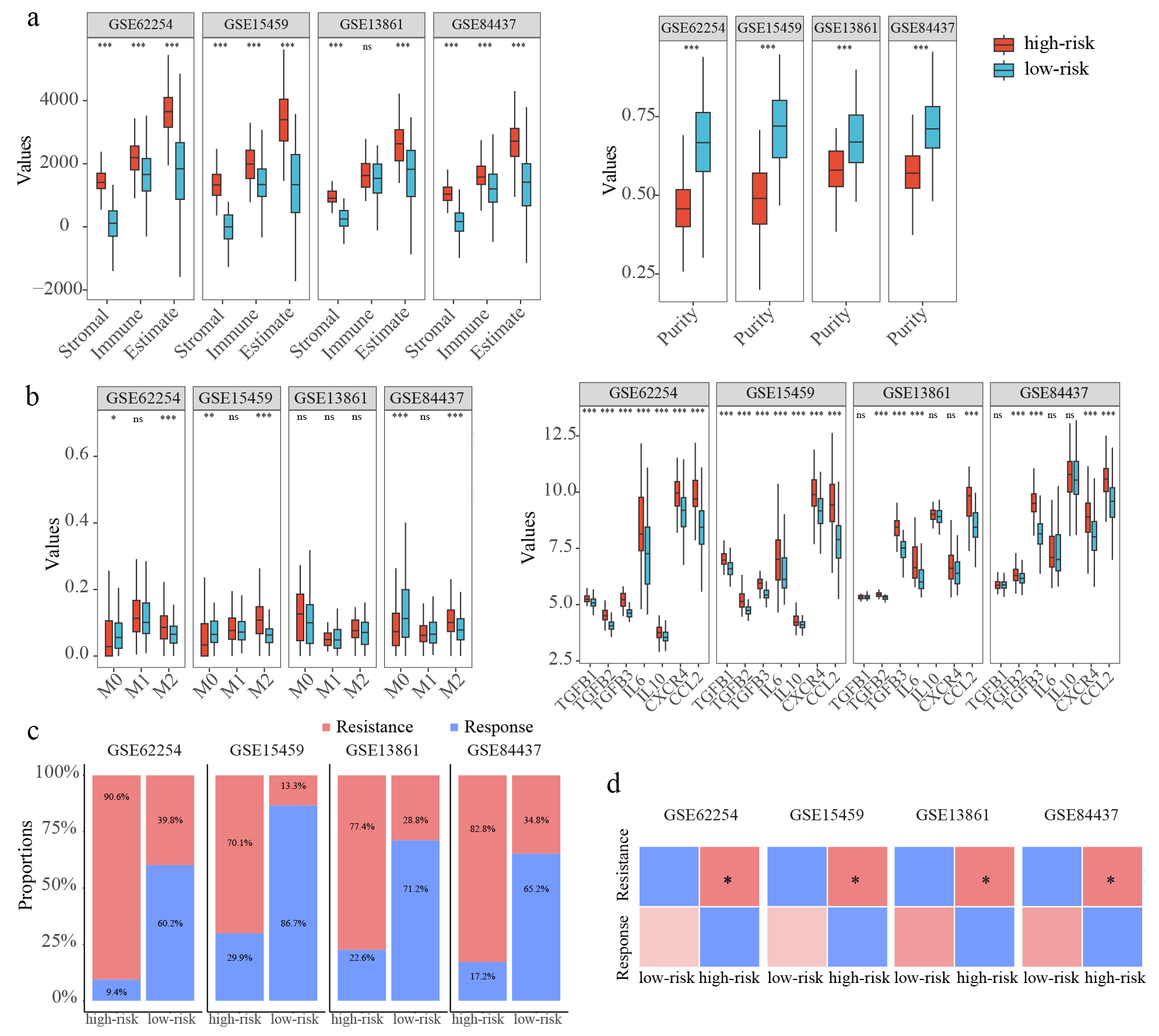
**

**Supplemental Fig. S9. The performance of 47-GPS in predicting immunotherapy responses. a** Comparison of the immune score, stromal score, ESTIMATE score and tumor purity between high-risk group and low-risk group in GSE62254, GSE15459, GSE13861 and GSE84437, respectively. **b** Comparison of infiltration densities of macrophage and expression of M2 polarization factor between high-risk and low-risk groups in GSE62254, GSE15459, GSE13861 and GSE84437, respectively. **c** TIDE analysis for samples in GSE62254, GSE15459, GSE13861 and GSE84437, respectively. Statistical signifcance: *P<0.05, **P<0.01, ***P<0.001. **d** Submap analysis for samples in GSE62254, GSE15459, GSE13861 and GSE84437, respectively.


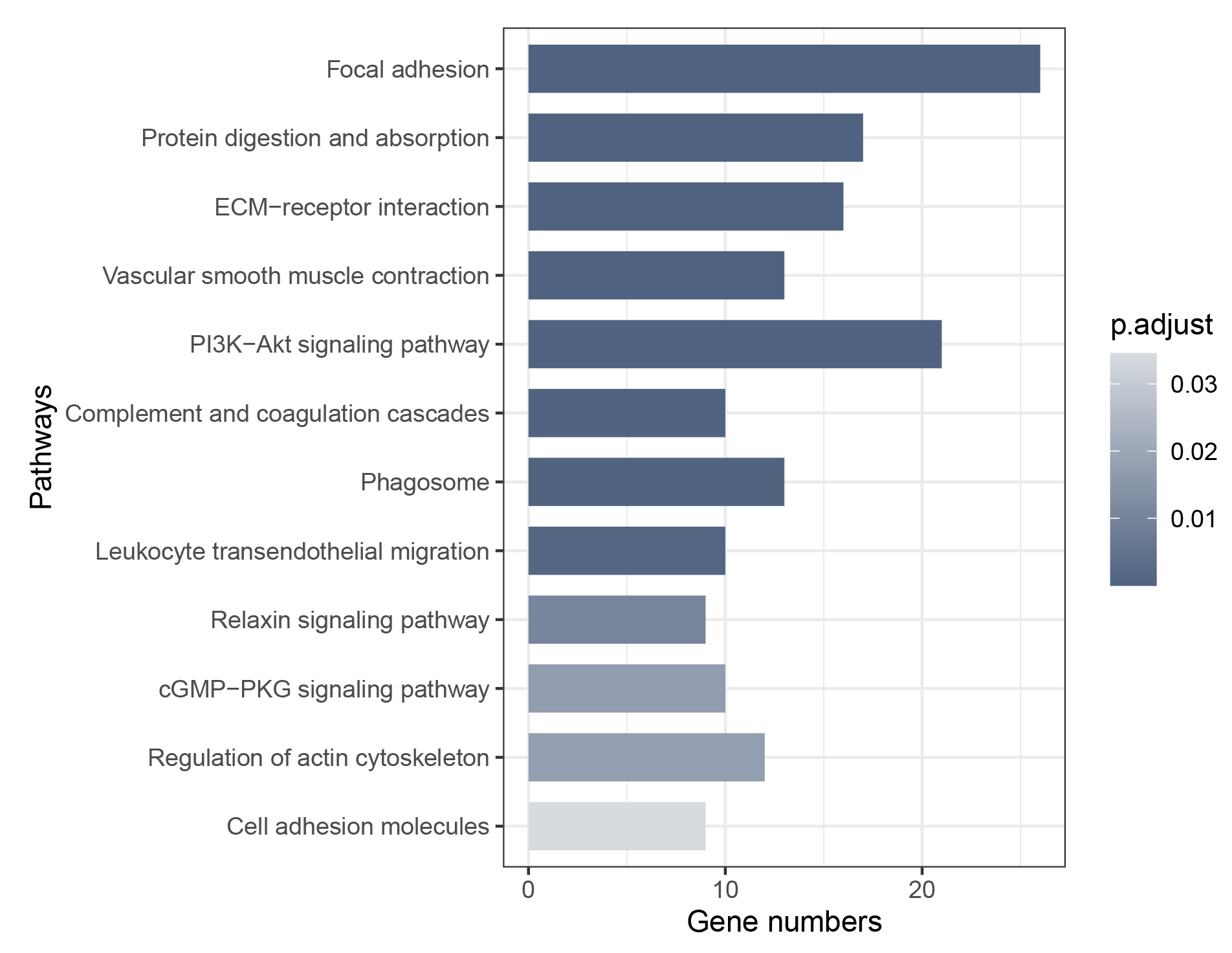
**Supplemental Fig. S10. The KEGG pathways enriched with the 359 uniformly up-regulated genes.**


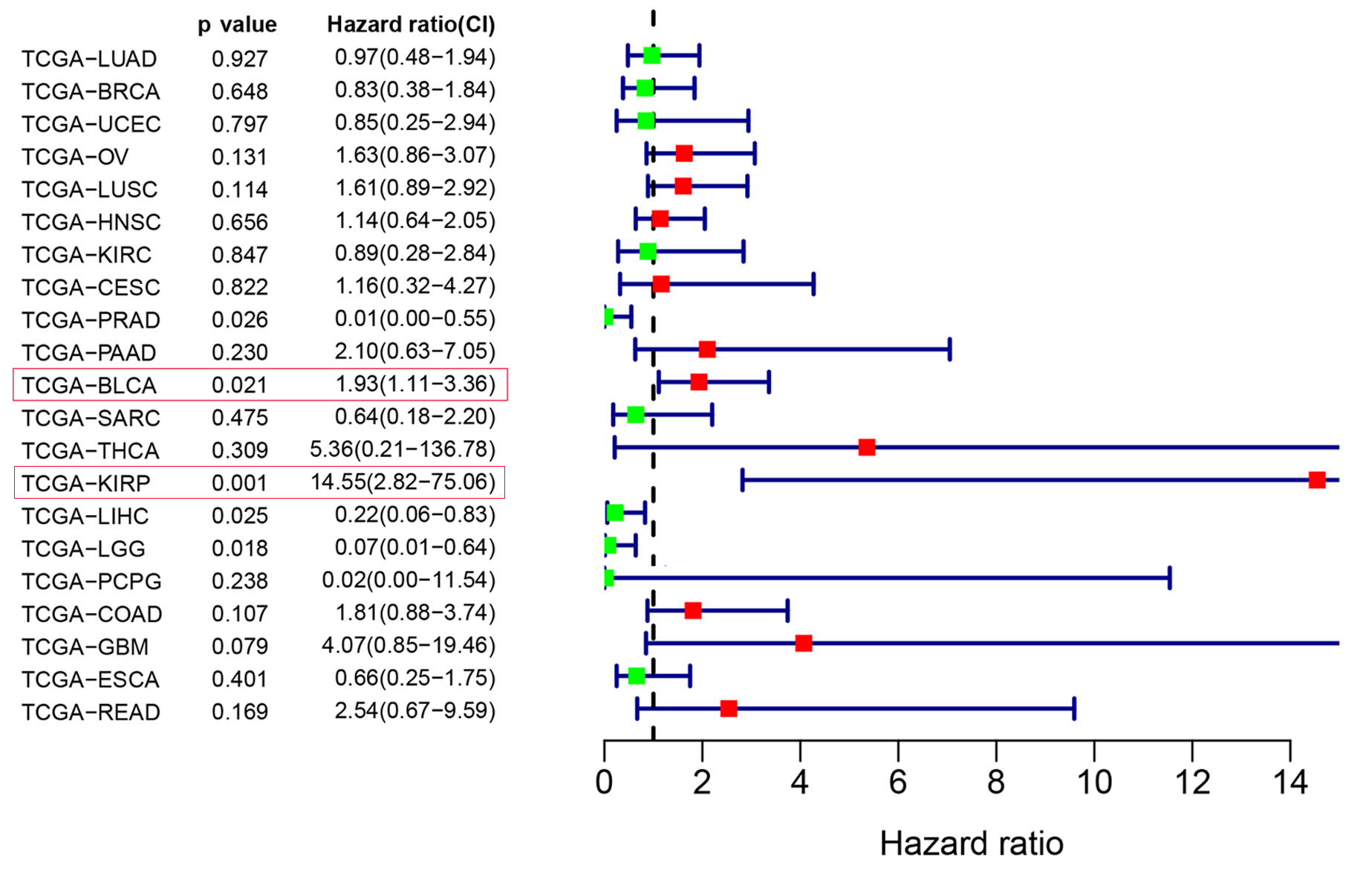
**Supplemental Fig. S11. The performance of 47-GPS in pan-cancer.**

**
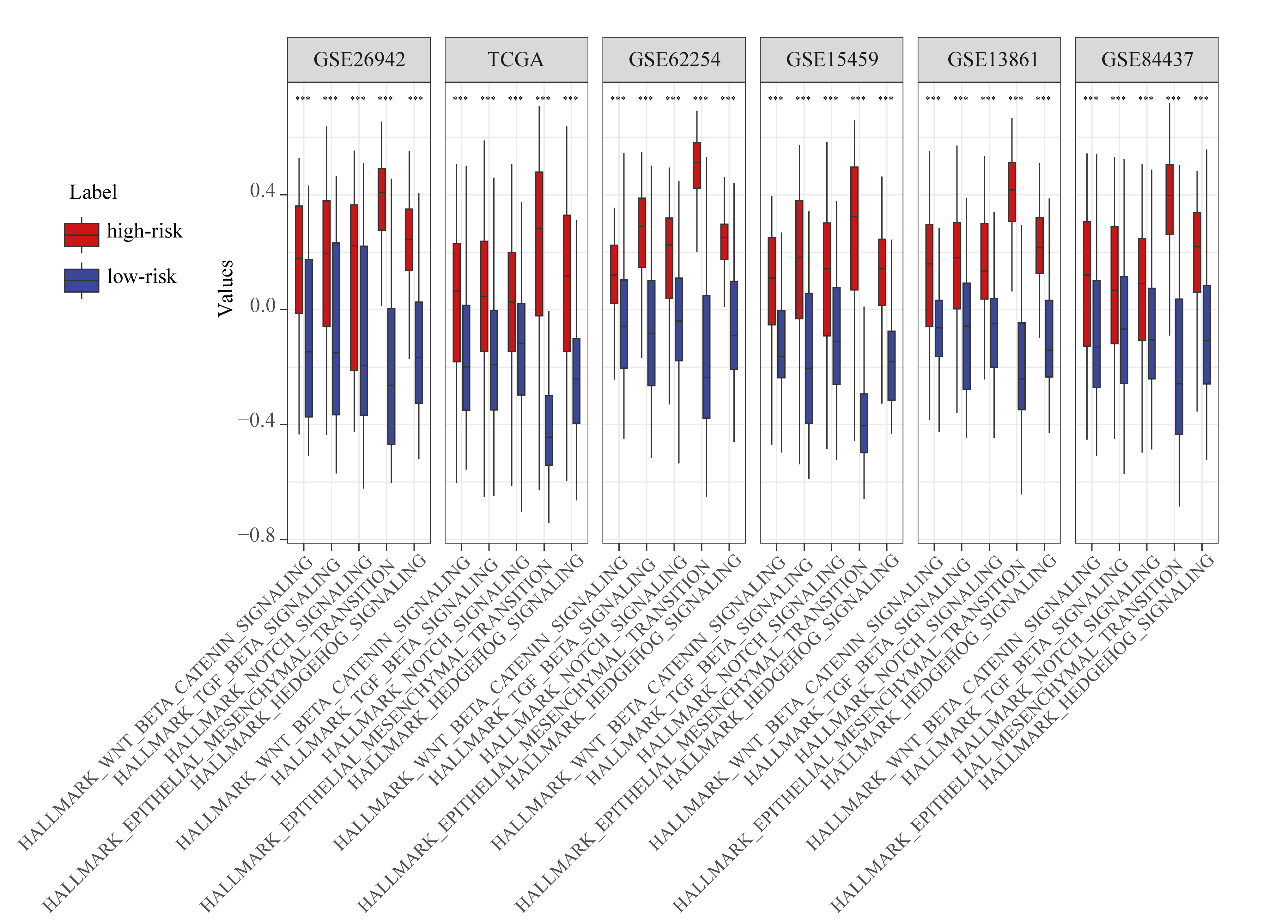
**

**Supplemental Fig. S12**. The enrichment scores between high-risk and low-risk groups in GSE26942, TCGA, GSE62254, GSE15459, GSE13861 and GSE84437, respectively.

**Supplemental data: Tables**

**Table S1. The 175 stemness-related signature genes.**

| Symbol | Symbol | Symbol | Symbol | Symbol | Symbol |
| --- | --- | --- | --- | --- | --- |
| FAM167B | CLEC2B | ID3 | PERP | MALL | APLNR |
| CNN3 | ARHGDIB | MYL12A | UPP1 | MORF4L1 | CCDC85B |
| IFI16 | CSRP2 | EID1 | ID1 | NPC2 | SERPINH1 |
| ACKR1 | ITM2B | CD59 | YBX3 | YPEL2 | NNMT |
| ABL2 | PCDH17 | CDC37 | PPDPF | ZFP36L1 | SLC2A3 |
| BMPR2 | ADCY4 | LGALS1 | KLF6 | TXN | GNAI2 |
| SH3BP5 | CRIP2 | SRP14 | KCTD12 | CTGF | LY6E |
| TGFBR2 | B2M | SERPING1 | JUN | PDLIM1 | SWAP70 |
| CLEC3B | MRTFB | WARS | EMP2 | LEPROT | TNFSF10 |
| HYAL2 | IGFBP4 | SNTB2 | SELENOW | ADAM15 | CTNNAL1 |
| ADAMTS9 | RAMP2 | CYSTM1 | RNASE1 | SEC62 | GSTP1 |
| ECSCR | SOCS3 | S100A6 | ANXA1 | IL6ST | UQCR10 |
| CD74 | TUBB6 | CARD16 | LAPTM4A | PSMA7 | HMGB1 |
| NRN1 | TCF4 | ARPC1B | C11orf58 | FEZ2 | ARL2 |
| RAMP3 | FKBP1A | TM4SF1 | ENY2 | KMT2E | CD99 |
| GNG11 | CD93 | CDC42EP3 | SLC25A5 | DNAJA1 | SERTAD1 |
| IL3RA | MADCAM1 | MTUS1 | LGALS3 | STAT3 | PRR13 |
| TSPAN7 | GADD45B | GABARAPL2 | PSMB8 | NCOA7 | ATP5MC3 |
| MSN | ICAM1 | RHOC | AGPAT2 | P4HB | FUS |
| ITM2A | JUNB | IRF1 | ATP1B3 | CYB5R3 | PLSCR1 |
| CLIC2 | BST2 | FXYD5 | IL32 | FOSB | PPA1 |
| SOX7 | PCAT19 | PSMB9 | ELOC | LAP3 | TRMT112 |
| DLC1 | BCAM | DUSP6 | MDK | RAB11A | TBCB |
| VIM | CLDN5 | CALM1 | PRXL2A | ISG20 | ELF1 |
| SNCG | JAM2 | S100A13 | ARL4A | UQCRH | COX8A |
| ENTPD1 | CCL2 | ECE1 | DUSP23 | TMEM50A | TSPAN4 |
| IFITM2 | KRT8 | TUBA1B | REG1A | TUBA1C | KRT18 |
| IFITM1 | LMCD1 | TSC22D1 | RAB13 | FLOT1 | TIMP1 |
| IFITM3 | IFI27 | EMP1 | PPIC | SERPINB1 | IER3 |
| - | - | - | - | - | PLK2 |

**Table S2. The C-index values of 47-GPS.**

| Pair | Gene1 | Gene2 | C-index |  | Pair | Gene1 | Gene2 | C-index |
| --- | --- | --- | --- | --- | --- | --- | --- | --- |
| 1 | VEGFC | RBM28 | 69.36% |  | 25 | ACKR1 | PHGDH | 71.21% |
| 2 | OLFML2B | XPO5 | 68.79% |  | 26 | PDGFRB | INPPL1 | 72.68% |
| 3 | CDH5 | MDC1 | 70.97% |  | 27 | CLEC14A | RPS6KB2 | 72.14% |
| 4 | MGP | WBP2 | 69.72% |  | 28 | CDH5 | ORC2 | 70.26% |
| 5 | PDGFRB | OSBP | 69.95% |  | 29 | MGP | SAE1 | 69.91% |
| 6 | NDN | SMUG1 | 70.74% |  | 30 | HSPA12B | SLX4 | 71.37% |
| 7 | LHFPL6 | FOXRED1 | 71.21% |  | 31 | THBS4 | TRAPPC6B | 71.82% |
| 8 | VEGFC | DBT | 71.29% |  | 32 | MGP | SRSF6 | 72.36% |
| 9 | SPARCL1 | MAT2A | 71.22% |  | 33 | SELP | TRMT10A | 72.55% |
| 10 | OLFML2B | FLYWCH2 | 70.60% |  | 34 | SFRP2 | TMEM126A | 72.72% |
| 11 | MGP | ALKBH5 | 70.94% |  | 35 | COL10A1 | EPS8L1 | 72.95% |
| 12 | FOXS1 | MCM10 | 70.36% |  | 36 | SFRP2 | ACP1 | 73.12% |
| 13 | TMEM204 | LRBA | 70.46% |  | 37 | COL10A1 | OTUD4 | 72.56% |
| 14 | DEPP1 | NOA1 | 69.99% |  | 38 | VEGFC | L3MBTL2 | 71.98% |
| 15 | SFRP2 | PDHA1 | 71.13% |  | 39 | ACKR1 | SLC25A51 | 72.56% |
| 16 | NRP1 | SEMA3B | 70.47% |  | 40 | PLPPR4 | WNT4 | 73.05% |
| 17 | SFRP2 | CNIH1 | 71.20% |  | 41 | NOD1 | XKR8 | 72.87% |
| 18 | HSPA12B | CEP164 | 70.12% |  | 42 | FSTL1 | HNRNPU | 72.96% |
| 19 | COL8A1 | SLC25A10 | 70.79% |  | 43 | SOX17 | OSGIN1 | 72.96% |
| 20 | COL10A1 | CBFA2T2 | 70.67% |  | 44 | VEGFC | PARPBP | 72.96% |
| 21 | MIR99AHG | FGA | 71.83% |  | 45 | COL10A1 | NSD3 | 72.85% |
| 22 | COL10A1 | RABIF | 71.68% |  | 46 | VEGFC | KIF14 | 72.85% |
| 23 | SPARCL1 | PPP1CC | 71.21% |  | 47 | SFRP2 | ARL1 | 74.00% |
| 24 | COL10A1 | CHD2 | 72.03% |  |  |  |  |  |

**Table S3. Consistency of differentially expressed genes between two datasets.**

| Dataset1 | Dataset2 | DEG1 | DEG2 | Intersection | Consistency |
| --- | --- | --- | --- | --- | --- |
| GSE13861 | GSE15459 | 746 | 2314 | 512 | 99.61% |
| GSE13861 | GSE26942 | 746 | 1909 | 630 | 99.84% |
| GSE13861 | GSE62254 | 746 | 2190 | 480 | 99.79% |
| GSE13861 | GSE84437 | 746 | 1515 | 520 | 99.81% |
| GSE13861 | TCGA | 746 | 1720 | 492 | 100.00% |
| GSE15459 | GSE26942 | 2314 | 1909 | 1139 | 100.00% |
| GSE15459 | GSE62254 | 2314 | 2190 | 1739 | 99.94% |
| GSE15459 | GSE84437 | 2314 | 1515 | 1013 | 100.00% |
| GSE15459 | TCGA | 2314 | 1720 | 1091 | 100.00% |
| GSE26942 | GSE62254 | 1909 | 2190 | 957 | 100.00% |
| GSE26942 | GSE84437 | 1909 | 1515 | 999 | 99.70% |
| GSE26942 | TCGA | 1909 | 1720 | 845 | 99.88% |
| GSE62254 | GSE84437 | 2190 | 1515 | 1003 | 100.00% |
| GSE62254 | TCGA | 2190 | 1720 | 1018 | 100.00% |
| GSE84437 | TCGA | 1515 | 1720 | 863 | 99.88% |

**Table S4. The summary of 19 genes.**

| Gene name | Summary |
| --- | --- |
| VEGFC | The protein encoded by this gene is a member of the platelet-derived growth factor/vascular endothelial growth factor (PDGF/VEGF) family. The encoded protein promotes angiogenesis and endothelial cell growth, and can also affect the permeability of blood vessels. The proprotein is further cleaved into a fully processed form that can bind and activate VEGFR-2 and VEGFR-3 receptors. |
| OLFML2B | This gene encodes an olfactomedin domain-containing protein. Most olfactomedin domain-containing proteins are secreted glycoproteins. |
| CDH5 | Functioning as a classical cadherin by imparting to cells the ability to adhere in a homophilic manner, this protein plays a role in endothelial adherens junction assembly and maintenance. |
| MGP | This gene encodes a member of the osteocalcin/matrix Gla family of proteins. The encoded vitamin K-dependent protein is secreted by chondrocytes and vascular smooth muscle cells, and functions as a physiological inhibitor of ectopic tissue calcification. |
| PDGFRB | The protein encoded by this gene is a cell surface tyrosine kinase receptor for members of the platelet-derived growth factor family. These growth factors are mitogens for cells of mesenchymal origin.This gene is essential for normal development of the cardiovascular system and aids in rearrangement of the actin cytoskeleton. |
| NDN | This intronless gene is located in the Prader-Willi syndrome deletion region. It is an imprinted gene and is expressed exclusively from the paternal allele. Studies in mouse suggest that the protein encoded by this gene may suppress growth in postmitotic neurons. |
| LHFPL6 | This gene is a member of the lipoma HMGIC fusion partner (LHFP) gene family, which is a subset of the superfamily of tetraspan transmembrane protein encoding genes. This gene is fused to a high-mobility group gene in a translocation-associated lipoma. Mutations in another LHFP-like gene result in deafness in humans and mice. |
| SPARCL1 | Predicted to enable calcium ion binding activity; collagen binding activity; and extracellular matrix binding activity. Predicted to be involved in anatomical structure development and regulation of synapse organization. |
| FOXS1 | The forkhead family of transcription factors belongs to the winged helix class of DNA-binding proteins. The protein encoded by this intronless gene contains a forkhead domain and is found predominantly in aorta and kidney. The function of the encoded protein is unknown. |
| DEPP1 | The expression of this gene is induced by fasting as well as by progesterone. The protein encoded by this gene contains a t-synaptosome-associated protein receptor (SNARE) coiled-coil homology domain and a peroxisomal targeting signal. Production of the encoded protein leads to phosphorylation and activation of the transcription factor ELK1. |
| SFRP2 | This gene encodes a member of the SFRP family that contains a cysteine-rich domain homologous to the putative Wnt-binding site of Frizzled proteins. SFRPs act as soluble modulators of Wnt signaling. Methylation of this gene is a potential marker for the presence of colorectal cancer. |
| COL8A1 | This gene encodes one of the two alpha chains of type VIII collagen. The gene product is a short chain collagen and a major component of the basement membrane of the corneal endothelium. The type VIII collagen fibril can be either a homo- or a heterotrimer. |
| COL10A1 | This gene encodes the alpha chain of type X collagen, a short chain collagen expressed by hypertrophic chondrocytes during endochondral ossification. Unlike type VIII collagen, the other short chain collagen, type X collagen is a homotrimer. |
| ACKR1 | The protein encoded by this gene is a glycosylated membrane protein and a non-specific receptor for several chemokines. The encoded protein is the receptor for the human malarial parasites Plasmodium vivax and Plasmodium knowlesi. Polymorphisms in this gene are the basis of the Duffy blood group system. |
| CLEC14A | This gene encodes a member of the C-type lectin/C-type lectin-like domain (CTL/CTLD) superfamily. Members of this family share a common protein fold and have diverse functions, such as cell adhesion, cell-cell signalling, glycoprotein turnover, and roles in inflammation and immune response. This family member plays a role in cell-cell adhesion and angiogenesis. It functions in filopodia formation, cell migration and tube formation. Due to its presence at higher levels in tumor endothelium than in normal tissue endothelium, it is considered to be a candidate for tumor vascular targeting. |
| THBS4 | The protein encoded by this gene belongs to the thrombospondin protein family. Thrombospondin family members are adhesive glycoproteins that mediate cell-to-cell and cell-to-matrix interactions. |
| SELP | This gene encodes a 140 kDa protein that is stored in the alpha-granules of platelets and Weibel-Palade bodies of endothelial cells. This protein redistributes to the plasma membrane during platelet activation and degranulation and mediates the interaction of activated endothelial cells or platelets with leukocytes. |
| PLPPR4 | The protein encoded by this gene belongs to the lipid phosphate phosphatase (LPP) family. LPPs catalyze the dephosphorylation of a number of bioactive lipid mediators that regulate a variety of cell functions. This protein is specifically expressed in neurons. It is located in the membranes of outgrowing axons and has been shown to be important for axonal outgrowth during development and regenerative sprouting. |
| FSTL1 | This gene encodes a protein with similarity to follistatin, an activin-binding protein. It contains an FS module, a follistatin-like sequence containing 10 conserved cysteine residues. This gene product is thought to be an autoantigen associated with rheumatoid arthritis. |
